# Memory profiles in Down syndrome across development: a review of memory abilities through the lifespan

**DOI:** 10.1186/s11689-017-9220-y

**Published:** 2018-01-29

**Authors:** Mary Godfrey, Nancy Raitano Lee

**Affiliations:** Department of Psychology, Drexel University, 3141 Chestnut Street, Stratton 119, Philadelphia, PA 19104 USA

**Keywords:** Intellectual disability, Cognition, Developmental trajectory

## Abstract

Down syndrome (DS) is associated with a variety of cognitive impairments, notably memory impairments. Due to the high prevalence rates of early-onset dementia associated with DS, it is imperative to understand the comprehensive development of memory impairments beginning in childhood and into adulthood, as this may help researchers identify precursors of dementia at earlier stages of development and pinpoint targets for memory intervention. The current paper provides a systematic, developmentally focused review of the nature of memory difficulties in DS across the lifespan. Specifically, this review summarizes what is known about long-term, short-term, and working memory abilities (distinguishing between verbal and nonverbal modalities) in DS, compared to both mental age-matched typically developing peers and individuals with other forms of intellectual disability (ID) at three developmental stages (i.e., preschool, adolescence, and adulthood). Additionally, this review examines the degree of impairment reported relative to typically developing mental age-matched peers in the existing literature by examining effect size data across memory domains as a function of age. With few exceptions, memory abilities were impaired across the lifespan compared to mental age-matched typically developing peers. Relative to other groups with ID, research findings are mixed. Our review of the literature identified a scarcity of memory studies in early childhood, particularly for STM and WM. In adulthood, research was limited in the LTM and WM domains and very little research has compared memory abilities in older adults with DS to those with typical development. Looking to the future, longitudinal studies could provide a better understanding of the developmental trajectory of memory abilities in DS, and the possible associations between memory abilities and real-world functioning. This research could ultimately inform interventions to improve independence and overall quality of life for those with DS and their families.

**Electronic supplementary material:**

The online version of this article (10.1186/s11689-017-9220-y) contains supplementary material, which is available to authorized users.

## Background

The current paper provides a systematic, developmentally focused review of the nature of memory difficulties in Down syndrome (DS) across the lifespan. We aim to add to and extend prior reviews (e.g., [1]) by describing what is known about different domains of memory function during different developmental periods. Additionally, to begin to examine memory impairments across the lifespan, we have calculated effect size estimates from published studies of long-term (LTM), short-term (STM), and working memory (WM) in which individuals with DS were compared to typically developing (TD) individuals matched on mental age (i.e., overall cognitive level). We have plotted these by domain (LTM, STM, WM), modality of the task (verbal, nonverbal), and mean chronological age of participants to begin to examine trends in findings and to identify developmental periods during which more research is needed. Our review is organized as follows: (a) we provide a brief summary of the DS cognitive phenotype to set the backdrop for interpreting memory impairments in DS; (b) we then describe neuropsychological conceptualizations of memory fractionation in order to organize the memory domains reviewed; and, finally, (c) we summarize past studies’ findings on LTM, STM, and WM in DS from preschool to adulthood.

DS is the most common form of intellectual disability (ID) with a specific genetic etiology, occurring in one of every 691 live births [2]. The syndrome results in a gradually declining IQ during childhood (i.e., cognitive gains do not keep pace with chronological age) [3]. Beginning at an early age, individuals with DS have impairments in adaptive functioning [4–6] and specific cognitive domains such as expressive language, and executive function that are in excess of overall cognitive impairments (for reviews, see [7–9]).

Furthermore, impairments in LTM are a prominent aspect of the DS cognitive phenotype and have been a particular area of research interest, in part due to the high prevalence of precocious-onset Alzheimer’s disease (AD) associated with DS. AD causes a progressive decline in cognitive abilities and functional skills and is the most common cause of dementia in the general population (for review, see [10]). The increased rates of precocious AD in DS are thought to be due in part to overexpression of genes on chromosome 21, most notably the amyloid beta precursor protein gene (for reviews, see [11–13]).

The neuropathology of AD is characterized by neuritic plaques and neurofibrillary tangles, which lead to death of neurons, brain atrophy, and cognitive decline [14]. Recent research on DS suggests that the amyloid beta accumulation first begins in the striatum, and progresses into the frontal lobes, and eventually the temporal lobe ([15, 16]; for review, see [12]). Consequently, those with DS and comorbid AD exhibit declines in executive function abilities, memory encoding and retrieval abilities, episodic memory and new learning abilities ([12, 17–19]). Although these symptoms of AD in DS are similar to symptoms in TD adults, the age of onset of AD within the DS population is much younger than in the TD population. By age 45, virtually all adults with DS have AD neuropathology; however, only 8.9% of adults with DS present with symptoms of AD at this age [20]. Nevertheless, AD symptom prevalence in DS is significantly higher than the AD symptom prevalence in typically developing (TD) adults, which occurs in only 0.003% of individuals at this age [21]. Moreover, by age 72, 67% of individuals with DS will meet the criteria for AD compared to 5% of TD adults [20, 22–24]. As a result, the development of memory impairments in adults with DS has been widely studied to better understand the risk factors for comorbid dementia.

Although understanding memory impairments in DS in middle and older adulthood has direct implications for understanding the development of comorbid AD, it is also important to understand memory impairments in DS across development. From infancy and childhood into adulthood, memory abilities are associated with adaptive behavior, independence skills, reading abilities, and general intelligence in DS [1, 9, 25, 26]. Thus, to provide strategic interventions, it is necessary to elucidate the relative strengths and weaknesses of specific memory domains across development. Furthermore, establishing a baseline of expected memory abilities across development could help clinicians identify impaired memory performance and subtle precursors to dementia earlier in development. Therefore, applying a developmental perspective to our conceptualization of the DS memory phenotype may provide crucial information necessary to improve clinical care and advance our theoretical models of memory development.

As argued by Karmiloff-Smith [27], utilizing a developmental approach to the study of cognition does not simply mean examining a pediatric sample, but rather it involves capturing changing abilities of a group over time. While the traditional neuropsychological “snapshot” approach of comparing a clinical group to a control group at one time point has provided valuable information about the DS cognitive phenotype, the trajectory approach allows us to map and track age and performance [28]. Therefore, this method promotes identification of developmental memory trends across the lifespan, indicating whether memory impairments increase or decrease with age.

The current review attempts to synthesize the existing literature on LTM, STM, and WM in DS within a developmental framework. However, it is important to note that the existing research literature on memory in DS does not permit the adoption of a traditional developmental trajectory approach to conceptualize changing memory impairments in DS across the lifespan. This is for two primary reasons. First, longitudinal studies of memory abilities are very limited for the group. Second, such an approach is best implemented when chronological age-matched comparison groups are included in studies as a benchmark of deviations from developmental expectations. Unfortunately, the inclusion of a chronological age-matched TD comparison group is a rarity in the literature on memory in DS. Rather, the vast majority of studies examining cognitive abilities of the DS population compare these to mental-age (MA)-matched peers because of the well-established understanding that cognitive functioning in DS is below chronological age expectations (for review, see [29]). Furthermore, the majority of neuropsychological assessments lack utility for a wide range of mental ages—that is, there are very few cognitive assessments that would challenge and maintain the attention of both an individual with DS and their chronologically age-matched peer. Due to this limitation, researchers studying DS have commonly used a MA comparison group. Therefore, this review of the literature will primarily focus on individuals with DS in comparison to MA-matched peers but will add a developmental perspective in that we will examine the degree of impairment relative to more general cognitive abilities as a function of the DS group’s chronological age.

The use of MA-matched peers as a comparison group is not only suboptimal for describing developmental trends relative to age expectations, it also makes comparing results across studies challenging, as different investigators may match on measures of overall cognitive ability, verbal cognitive ability, or nonverbal cognitive ability. For a group like DS with pronounced impairments in some aspects of language functioning (for a review, see [30]), matching on verbal MA may result in a pattern of findings in which nonverbal memory impairments look smaller than they would if groups were matched on nonverbal cognitive ability. In this latter scenario, that is matching on nonverbal cognitive abilities, impairments on verbal memory tasks may appear larger or be exaggerated due to the nonverbal cognitive matching strategy. Thus, in order to be clear about the nature of the existing findings in the literature, our review, including summary tables and figures, which will be described later, includes details about the matching strategy employed. We hope that this will aid the reader in interpreting the existing findings and thus trends in memory abilities relative to overall cognitive abilities across development in DS. Prior to describing this literature, we will provide a brief overview of neuropsychological conceptualizations of memory fractionation to provide a framework to organize the memory domains included in the current review, as well as a justification for the choice of memory domains reviewed.

## Neuropsychological conceptualizations of memory

Current conceptualizations of retrospective memory, the process of recalling previously learned information, divide this construct into two forms: implicit and explicit memory [31]. Implicit memory involves retrieving information, without conscious awareness, to perform an action. It is thought to rely upon the basal ganglia [32–34] and broader cortico-striatal networks (see [31, 35] for reviews). This review will not examine implicit memory, due to the limited number of studies examining this construct in DS. Furthermore, existing studies suggest that implicit memory tends to be MA appropriate in DS. For example, Vicari and colleagues ([36, 37]) reported comparable performance on implicit memory tasks between those with DS and typically developed MA-matched children. Moreover, they reported stronger implicit memory performance in DS compared to ID peers (e.g., Williams syndrome). Consistent with these findings, Bussy and colleagues [38] reported that those with DS could implicitly learn a sequence of movements at levels comparable to MA-matched TD peers. Thus, the limited research available indicates that while implicit memory is not equivalent to chronological age expectations in adolescents and adults with DS, it is not impaired beyond mental age expectancies in DS. Because past research with children with DS consistently has found impairments in explicit memory that exceed global learning difficulties (i.e., performance below MA expectations) [36, 37], we will focus our review on explicit memory systems.

Explicit memory consists of actively retrieving memories and being cognizant of the prior learning process. Explicit memory can be categorized as either semantic memory (i.e., general knowledge, facts, and vocabulary) or episodic (i.e., biographical, personal events, contextual memories) [39]. Furthermore, memory models categorize the construct by time length and quantity of information to be recalled. With regard to time length, models have traditionally distinguished memory as either upheld for a matter of seconds (i.e., STM) or stored and retrieved at a later point in time (i.e., LTM) [40, 41]). These distinctions have roots in James’ early descriptions of primary (STM) and secondary (LTM) memory ([42]). Another factor that has been used to distinguish STM and LTM is the amount of information to be recalled [43]. More specifically, STM is believed to have a limited capacity, that is, the to-be-recalled information must fall within an individual’s immediate memory span [43, 44]. Tasks that exceed an individual’s immediate memory span are referred to as supraspan memory tasks and are often categorized under the LTM umbrella. For the purposes of the current paper, we will refer to tasks that require *immediate* recall of *subspan* stimuli as *STM* tasks and those that require the recall of *supraspan* stimuli *or impose a delay* prior to the testing of recall as *LTM* tasks.

In addition, memory models often include WM (i.e., recalling and manipulating information to complete some task). Although both WM and STM involve short-term storage, models distinguish WM from STM due to the increased processing demands associated with WM tasks (i.e., attending to and manipulating information) [44, 45]. This distinction appears to be an important one when considering relations between these two short-term memory systems and higher level cognitive skills. Specifically, research suggests that WM, in particular, is highly predictive of intellectual abilities in those with typical development [46]. Furthermore, research has suggested a discrepancy between STM and WM abilities in the DS population. Individuals with DS demonstrate a significant impairment in verbal and nonverbal WM skills, yet do not consistently demonstrate impairment in the nonverbal STM domain despite profoundly impaired verbal STM abilities (for a review, see [47]). Consequently, to better understand the nuanced memory abilities of individuals with DS, we will examine WM distinctly from STM.

Lastly, it should be noted that while we have organized our review of memory in DS utilizing these three memory domains, theories of memory and its fractionation are ever evolving, with some researchers arguing that the content of memory is more important to consider than the timing of stimuli presentation and recall (e.g., [48]). Given that the vast majority of studies examining memory in DS utilize LTM, STM, and WM to describe tasks demands, the current review adopts these terms. However, we will briefly touch upon alternative memory frameworks and their implications for the future of DS memory research in the “Discussion” section.

We will now review what is known about LTM, STM, and WM abilities in DS over the lifespan. Our review of these memory domains will also distinguish between impairments in verbal and nonverbal modalities (i.e., visual or spatial abilities), as memory abilities do appear to differ based on the modality of the to-be-recalled stimuli ([49]; for review, see [47]). Additionally, memory studies will be organized into three developmental periods: preschool (≤ 5 years old), school-age and adolescence (6 to 17 years old), and adulthood (≥ 18 years).

## Literature review and analytic methods

The current literature was garnered through a systematic search of online databases. Specifically, keyword combinations included “Down Syndrome” or “Trisomy 21” and “memory,” “short-term memory,” “long-term memory,” and “working memory”. This search produced 1534 unique articles via the online databases PubMed, PsycInfo, and Web of Science. (A total of 2446 articles were identified with 912 redundancies.)

The first author completed the initial search of the literature. This was followed by a second review of abstracts by the second author. Studies were selected that either (a) compared a participant group with DS to a control group (either typically developing individuals or individuals with intellectual disabilities) or (b) examined older adults with DS and compared performance at different ages. To be included in the current review, the study was required to include at least one memory assessment (i.e., LTM, STM, or WM). This resulted in a selection of 106 studies which have informed our review of LTM, STM, and WM in DS.

**Table 1:** Studies Examining LTM Skills in Individuals with DS Organized by Age Group and Presented Chronologically

Study	Comparison Group	Matching Assessment	CA of DS group (Yrs)	MA of DS group (Yrs)	Task	Findings
**Preschool studies (< 5 years old)**						
*Milojevich 2015* [54]	MA-matched TD	Bayley- III	M = 2.75 (Range 1- 4)	M = 0.9	*Visual LTM* Deferred Imitation	DS ~ TD-MA on number of target actions after 1 month delay DS < TD-MA temporal sequence of actions after 1 month delay TD-MA: Familiar target actions > Novel target actions DS: Familiar target actions ~ Novel target actions
*Roberts 2015* [35]	Verbal MA-matched TD	MSEL (Verbal scales)	M= 4 (Range 3-5)	M = 2.8	*Visual LTM* Deferred Imitation Task Object Location Retention task	DS > TD-Verbal MA DS ~ TD-Verbal MA (Results same when groups matched on nonverbal ability)
**School Age and Adolescent studies (6 to 17 years old)**						
Carlesimo 1997 [56]	ID group MA-matched TD	WISC-R or WAIS	M=16	M = 9.1	*Verbal and Visual LTM* Word List Learning Story Recall Word Recognition Rey Figure Form B	DS < ID < TD-MA on immediate & delayed recall DS < ID < TD-MA DS < TD-MA; DS~ID on hit rate and false alarms DS < TD-MA; DS~ID on immediate & delayed recall
Mattson 1999 [59]	TD & FAS	--	M=12.9 (Range = 9.3-16.0)	--	*Verbal LTM* Word Recall Word Recognition	DS < FAS < TD DS < FAS < TD
Dawson 2001 [157]	MA - matched TD & ASD	Preschool Language Scale-3	M=5.7	M=2.5	*Visual LTM* Visual Paired Comparison (5-minute delay)	DS ~ TD-MA DS ~ ASD
Byrne 2002 [60]	Reading Matched & Average TD Readers	BAS Word Reading	M=8 (Range= 4-12)	M=6.3	*Visual LTM* Delayed Visual Recall	DS < TD-RM and TD-AR *All 3 groups showed steady progress in their memory abilities. Groups did not significantly differ in amount of progress made over the two years
*Pennington 2003* [9]	MA-matched TD	DAS	M=14 (Range=11-19)	M=4.5	*Verbal, Spatial, Visual LTM* List Learning Maze Learning task Pattern Recognition Paired Associates Learning Ecological Memory task	DS < TD-MA DS < TD-MA DS < TD-MA DS < TD-MA DS < TD-MA on some aspects of task; DS~TD-MA on other aspects
Nichols 2004 [57]	MA-matched TD SLI WS Focal Lesions	WISC-R or WAIS-R	M=15.0	M=7.9	*Verbal LTM* CVLT-C	Long Delay Free Recall: DS<TD-MA; DS~WS, SLI Number of Intrusions: DS > TD-MA, FL Recognition Memory/Discriminability: DS < TD-MA; DS~WS, SLI
Bird 2004 [158]	NVMA-matched TD	SB	M=16.4 (Range=12 -20)	M=5.4	*Verbal LTM* Immediate & Delayed Story Recall (with embedded novel, nonsense words)	DS<TD-NVMA on immediate not delayed verbatim story recall (which included nonsense words)
*Vicari 2005* [62]	MA-matched TD	SB	M=16 (Range=8-30)	M=5.3	*Visual and Spatial LTM* Visual-Object Learning Visual-Spatial Learning	DS < TD-MA DS ~ TD-MA
Jarrold, 2007 [55]	WS group TD	Not matched	M=13 (Range=10-16)	M=5.3	*Verbal and Visual LTM* People Test – verbal recall Shapes Test – visual recall	DS ~ TD DS < TD; DS~WS
*Visu-Petra 2007* [120]	MA-matched TD	SB	M=14 (Range=8-21)	M=5.75	*Visual & Spatial LTM* Delayed Match to Sample Paired Associates Learning Number of Stages Completed Spatial Recognition Memory	DS < TD-MA DS < TD-MA DS < TD-MA
Edgin 2010 [25]	IQ-matched WS group	WASI	M=17.8	--	*Verbal and Visual LTM* NEPSY List Learning CANTAB Paired Associates Number of Stages Completed	DS > WS DS > WS
Heimann 2016 [61]	TD, Language- matched ASD	--	M=5.25 (SD=1.03)	M=2.63 (SD=.72)	*Visual LTM* Deferred Imitation	DS ~ TD DS > ASD
**Adult and Older adult studies (18 years and older)**						
Dalton 1974 [159]	Young and Old ID group	--	Young M= 20.9 Intermediate M=41.7 Old M= 50.8 (Range=19-58)	--	*Visual LTM* Delayed Matching to Sample	Young DS < Young & Old ID; Young DS = Intermediate DS Intermediate DS < Old ID; Intermediate DS = Young ID Old DS < all other DS and ID groups
Varnhagen 1987 [64]	IQ-matched ID group	SB	M=22.70 (Range=19-25)	M=4.78	*Verbal LTM* Lexical Access – a measure of lexical LTM access derived from verbal measures	DS < ~ ID (p=.06)
Ellis 1989 [160]	TD group (matched roughly on CA)	--	M=26.8 (Range=14-51)	--	*Visual LTM* Picture Recall Picture Location Recall	DS < TD college students DS < TD college students
Devenny 1992 [161]	ID < 35 ID > 35	-	Young DS M=31.7 Old DS M=41.8	-	*Verbal LTM* Selective Reminding Test	Both TD and DS participants showed no significant change in performance from Time 1 to Time 2
Brugge 1994 [63]	CA-matched ID group	--	M=31 (Range=22-51)	--	*Verbal LTM* CVLT	DS<ID on various scores CVLT Short Delay Savings score predictive of group. DS performance inversely related to age
Simon 1995 [70]	IQ-matched ID & CA-matched TD	--	M=30	--	*Verbal and Visual LTM* Word Recall Facial Recognition Picture Recall	DS < TD-CA; DS~ID DS ~ ID; DS<TD-CA DS < ID & TD-CA
Devenny 1996 [65]	Young DS (CA 30-39) Middle-Aged DS (CA 40-49) Old DS (CA 50+) ID group with similar IQ & Age	Subtests of WISC-R were used to estimate IQ	Young DS M=37 Middle-Aged DS M=45 Older DS M=55	-	*Verbal LTM* Selective Reminding Test (SRT)– word recall	*Participants were tested at 6 time points across 5 years* DS: Less improvement than ID on repeated testing Young DS ~ Improvement on SRT across time points Middle-Aged and Older DS ~ Decrease in SRT across time points *Note: This study focused on a more mildly impacted subgroup of adults with DS
Dulaney 1996 [71]	CA- matched TD; ID	--	M=33.6 (SD=6.8)	--	*Visual & Spatial LTM* Picture Recognition Spatial Location Recall of pictures	DS < TD-CA; DS ~ ID DS < TD-CA; DS ~ ID
*Vicari 2000* [36]	MA-matched TD	SB	M= 21	M=6.5	*Verbal and Visual LTM* Word Recall Picture Recognition Spatial Sequence Learning	DS <TD-MA on word recall; DS > TD-MA on false alarms DS < TD-MA on picture recognition; DS > TD-MA on false alarms DS > TD-MA on number of trials to learn sequence *higher number of trials indicates worse performance
Krinsky-McHale 2005 [162]	IQ Matched WS ID		M=44.36 (SD=5.56)		*Verbal LTM* Selective Reminding Task	For both DS and WS, number of words recalled decreased with age; not observed in ID group
*Davis 2014* [67]	MA-matched TD and ID	Leiter-Revised Brief Form & KBIT-2	M~18	M=5.0	*Spatial LTM* Route Learning Recall	DS< TD-MA & ID
Purser 2015 [69]	WS group TD group (similar mental age)	--	M=18 (Range=10-39)	--	*Visual Spatial LTM* Route Learning Task – Errors	DS > TD & WS (errors)
Lavenex 2015 [68]	NV MA-matched TD	Leiter-Revised	M=18.8 (Range=11-29)	M=5.3	*Spatial LTM* Allocentric Spatial Memory Task	DS<TD-MA
**Within DS Group Comparisons of Older Adults**						
Haxby 1989 [163]	35+ DS (4 with dementia) Under 35 DS	--	Under 35 M=26 35+ non-demented M=48 35+ demented M=57	Under 35 M= 6.1 35+ non-demented M=4.3 35+ demented M=2.5	*Visual LTM* Hidden Object Memory Recognition for Designs	35+ Non-demented DS < Under 35 DS; demented DS not compared All 35+ DS (demented & non-demented) < Under 35 DS
Alexander 1997 [66]	Young DS (CA 22-38) Old DS without dementia (CA 41-61)	--	Young DS M=30.1 Old DS M=47.4	Young DS M=5.7 Old DS M=4.7	*Visual LTM* Hidden Object Memory (10-second delay) Hidden Object Memory (2-minute delay)	Old DS < Young DS Old DS < Young DS
Crayton 1998 [72]	Young DS (CA Under 40) Middle-Aged DS (CA 40-49) Older DS (CA 50+)	--	--	M =4.8 (for whole group on BPVS)	*Visual and Spatial LTM* Pattern Recognition Spatial Recognition Delayed Associative learning	Young DS ~ Middle-Aged DS ~ Older DS Middle Aged DS < Young DS Older DS < Young DS
Hon 1998 [73]	Young DS (CA 30-44) Old DS (CA 45-65) - 10 with AD	--	Overall M=42.6	--	*Verbal and Visual LTM* Rivermead - Name Learning Rivermead - Face Learning Rivermead - Route Learning Rivermead- Story Recall	Old DS < Young DS Old DS < Young DS Old DS < Young DS Old DS < Young DS
Dalton 1999 [164]	Young DS (CA 17-39) Old DS (CA 40-58)	--	--	--	*Visual LTM* Matching to Sample (25 second delay)	Old DS < Young DS
Krinsky-McHale 2002 [18]	DS with dementia (DS+DAT) DS without Dementia (DS)		*DS+DAT* Females M=52.23 Males M-45.32 *DS* Females M=42.06 Males M=44.36	--	*Verbal LTM* Selective Reminding Test (word list recall)	DS+DAT greater decline in performance over 3 years relative to DS Older DS+DAT & DS greater decline in performance over 3 years than Younger DS & DS+DAT
Devenny 2002 [165]	--	--	DS no DAT=47.3, DS DAT= 54.8	--	*Visual LTM* Cued Recall Task	DS DAT < DS no DAT

Key: Comparison Groups: *CA* chronological age, *DAT* Dementia of the Alzheimer’s Type, *DS* Down Syndrome, *ID* Intellectual Disability, *MA* mental-age, *NVMA* Nonverbal mental-age, *RA-matched* reading age matched, *SLI* Specific Language Impairment, *TBI* Traumatic Brain Injury, *TD* typically developing, *WS* William’s Syndrome

Tests: *BAS* British Abilities Scale, *Bayley* Bayley Scales of Infant Development, *BPVS* British Picture Vocabulary Scale, *CANTAB* Cambridge Neuropsychological Test Automated Battery, *CVLT-C* California Verbal Learning Testing Children’s Version, *DAS* Differential Ability Scales, *K-BIT* Kaufman-Brief Intelligence Test, *Leiter* Leiter International Performance Scale, *MSEL* Mullen Scales of Early learning, *RCPM* Raven’s Coloured Progressive Matrices, *Rivermead* Rivermead Behavioral Memory Test for Children, *SB* Stanford Binet Intelligence Scale, *WAIS-R* Wechsler Adult Intelligence Scale-Revised, *WASI* Wechsler Abbreviated Scale of Intelligence, *WISC-R* Wechsler Intelligence Scale for Children- Revised

*Note*: Italicized authors’ names indicate studies included in effect size analyses

**Table 2:** Studies Examining STM Skills in Individuals with DS Organized by Age Group and Presented Chronologically

Study	Comparison Group	Matching Assessment	CA of DS Group (Yrs)	MA of DS Group	Tasks	Findings
Valencia-Naranjo 2017 [83]	CA & MA-matched FXS	K-BIT	M=4.29	--	*Verbal and Visual STM* ^*1*^ Story Recall Object Recall	DS< FXS DS< FXS
**School Age and Adolescent Studies (6 to 17 years old)**						
Study	Comparison Group	Matching Assessment	CA of DS Group (Yrs)	MA of DS Group	Tasks	Findings
Dodd 1975 [166]	ID	--	M=9.1	--	*Verbal STM* Real & Nonsense Word Discrimination (0, 15, and 30 second delays)	DS>ID - Recognition on 0 and 30 sec delay; DS<ID Recall on 15 and 30 sec delay
McDade 1980 [167]	CA-matched TD MA-matched TD	SB	M~10	Range= 3.5 – 5.5	*Verbal STM* Digit Span Recall Verbal Recognition Test (forced choice recognition on a word recall task)	DS < TD-MA < TD-CA DS < TD-CA; DS ~ TD-MA
Strattford 1982 [168]	TD & ID	SB & PPVT	M=10.6 (SD=3.92)	M=4.9	Visual STM Immediate design reproduction	DS < TD; DS~ID
Snart 1982 [169]	ID group & IQ-matched TBI	SB WISC-R	M=16.3 DS & control (Range = 9-22)	--	*Verbal STM* Auditory Serial Recall	DS< ID and TBI on successive auditory recall factor score
Marcell 1988 [170]	VMA-matched TD ID group	PPVT	M=16.75	M=4.89	*Verbal STM* Digit Span – Auditory Freefield (traditional) Auditory presentation with headphones Auditory presentation with participants wearing goggles (to reduce distraction) *Visual/Verbal STM* Digits presented visually, recalled orally	DS< TD-MA, ID DS< TD-MA, ID DS< TD-MA, ID DS~ TD-MA, ID
Bower 1994 [88]	IQ and CA matched ID	--	Range=5-18	M=4.6	*Verbal STM* Digit Recall Sentence Recall	DS < ID DS < ID
Kay-Raining 1994 [171]	MA-matched TD	SB	M=14		*Verbal STM* Digit Span Recall	DS < TD-MA
Wang 1994 [89]	IQ & CA matched WS group	WISC-R	M=15.4	--	*Verbal & Spatial STM* Digit Span Corsi Block Recall	DS < WS DS > WS
Cornish 1999 [95]	CA-matched TD, MA-matched TD, FXS	--	M=10.82 (SD=1.89)	M=5.10 (SD=1.21)	*Visual STM* K-ABC Spatial Memory Task	DS < FXS, TD-CA& TD-MA
*Munir 2000* [58]	MA-matched TD, CA-matched TD and FXS	BPVS	M= 11.17 (2.49)	M=6.09	*Verbal & Visual STM* Children’s Nonword Repetition Task WISC-III Forward Digit Span Kaufman Spatial Location Recall	DS >FXS; DS ~ TD-MA & TD-CA DS ~ FXS; DS <TD-MA & TD-CA DS > FXS; DS < TD MA & TD-CA
*Seung 2000* [26]	NVMA-matched TD MLU controls	SB NV subtests for NVMA TD group	M=16.4 (Range=9-24)	M=5.58	*Verbal STM* ITPA Forward Digit Span Length	DS < TD-NVMA DS ~ MLU
Kanno 2002 [172]	TD (with similar MA)	--	M=16.10 (SD=1.64)	M=6.02	*Verbal STM* Serial Recall of words	DS < TD-MA
*Jarrold 2002* [49]	ID group VMA-matched TD	BPVS	M=14.28 (Range=8-17)	M=5.09	*Verbal & Spatial STM* Digit Span Recall +/- visual support Corsi Span Recall	DS < Combined ID & TD-VMA comparison group DS ~ Combined ID & TD-VMA comparison group
*Laws 2002* [147]	VMA-matched TD	BPVS-II	M=11.1 (Range=7-17)	M=4.0	*Verbal, Visual, & Spatial STM* Digit Span Recall Corsi Span Recall Color Memory	DS < TD-VMA DS > TD-VMA DS< TD-VMA on focal colors DS ~ TD-VMA on non-focal colors
Byrne 2002 [60]	Reading Matched TD Average readers TD	BAS Word Reading	M=8	M=6.3	*Verbal & Visual STM* BAS Digit Recall BAS Immediate Visual Recall	DS < TD-RM < TD-AR DS < TD-RM & TD-AR
Eadie 2002 [127]	SLI group TD group matched on MLU	--	M=7.2	--	*Verbal STM* Sentence repetition	DS < TD-MLU; DS ~ SLI
*Pennington 2003* [9]	MA-matched TD	DAS	M=14 (Range=11-19)	M=4.5	*Verbal STM* Digit Recall	DS <TD-MA
Vicari 2004 [96]	MA-matched TD; MA & CA matched WS	SB	M=13.4 (SD=4.4)	M=5.2	*Verbal & Visual STM* Digit Forward Span Test Spatial Forward Span Test	DS < WS; DS < TD-MA DS ~ WS; DS < TD-MA
*Lanfranchi 2004* Study 1 [115]	MA-matched TD	Logical Operations Test	M=11.75 (Range = 7-16)	M=5.42	*Verbal STM* Word Span Recall	DS < TD-MA
*Lanfranchi 2004* Study 2 [115]	MA-matched TD	Logical Operations Test	M=14.5 (Range= 11-18)	M=4.5	*Visual STM* Memory for Positions Pathway Forwards (recall of sequenced moves)	DS ~ TD-MA DS ~ TD-MA
Seung 2004 [174]	MA-matched TD, MLU	--	M=17.1 (SD=4.4)	M=5.8	*Verbal STM* SB Sentence memory subtest	DS < TD MA DS ~ MLU
Fidler 2005 [173]	NV IQ-matched ID	K-BIT	M=13.25 (Range=7-21)	---	*Verbal STM* Number Sequential Recall	DS<ID
Brock 2005 [175]	TD group Groups similar on BPVS, though not explicitly matched	--	M=17.6 (Range=8-25)	M=7.1	*Verbal STM* Digit Span recall Digit Reconstruction recall (pointing to numbers)	DS<TD DS<TD
*Cairns 2005* [176]	VMA-matched TD	BPVS	M=15.43 (Range=12-19)	M=5.16	*Verbal STM* Word & Nonword Repetition Digit Span Recall	DS<TD-VMA DS<TD-VMA
Hick 2005 [177]	NVMA-matched SLI NVMA-matched TD	Leiter	M=9.75	M=4.5	*Verbal & Visual STM* Digit Span Recall Word Span Recall Pattern Recall	DS < TD-NVMA, DS ~SLI DS < TD-NVMA, DS ~ SLI DS~SLI~TD-NVMA Verbal STM Tasks – SLI made progress over time that was not seen in DS
Vicari 2006 [178]	MA-matched TD	SB	M=15.83 (SD=5.66)	M=5.16	*Visual & Spatial STM* Spatial Location Span Visual Span	DS < TD-MA DS < TD-MA
*Visu-Petra 2007* [120]	MA-matched TD	SB	M=14 (R=8-21)	M=5.75	*Spatial STM* CANTAB Spatial Span Task	DS ~ TD-MA (TD-MA> DS trend)
Keller-Bell 2007 [179]	NVMA-matched TD	Columbia Mental Maturity Scale	M=9.40 (Range=5-12)	M=4.65	*Verbal STM* Nonword Repetition task	DS < TD-NVMA
Lanfranchi, 2009 [142]	MA-matched TD	PPVT-Revised and RCM	M=12.5 (Range=7-17)	M=6.0	*Visual STM* Pathway Recall Positions Recall	DS ~ TD-MA DS < TD-MA
*Frenkel 2009* [87]	MA-matched TD	K-ABC	M=12 (Range=6-17)	M=5.01	*Verbal, & Spatial STM* Auditory Word Span Task Visual Patterns Task Corsi Block Recall Task	DS < TD-MA DS < TD-MA DS ~ TD-MA
Kogan 2009 [97]	FXS group VMA-matched TD CA-matched TD	PPVT- III	M=17.16 (Range=11-36)	M=6.35	*Visual & Spatial STM* Delayed-non-matching-to-sample Delayed-non-matching-to-position (spatial)	DS~FXS~TD-VMA DS ~ FXS; DS < TD-CA
Cardoso-Martins 2009 [180]	MA-matched TD	DAS	M=14.48	--	*Verbal & Spatial STM* Digit Span Corsi Blocks Recall	DS< TD-MA DS~TD-MA
*Lanfranchi 2009* [117]	VMA matched TD Verbal matched TD	PPVT-R and WPPSI Verbal	M=13.1 (Range=8-19)	M=4.11	*Verbal & Spatial STM* Word Span Path Recall	DS< TD-VMA & TD-verbal matched DS~TD-VMA & TD-verbal matched
Abdelhameed 2010 [181]	MA-matched TD	SB-4	M=9.94 (Range=7-13)	M=3.94	*Verbal STM* Digit Span Recall Nonword Repetition	DS < TD-MA DS < TD-MA on all syllable lengths
Carretti 2010 [182]	MA-matched TD	PPVT	M=7.6 (SD=2.2)	M=5.2	*Visual STM* Spatial Recall – Structured Pattern Spatial Recall – Random Pattern	DS < TD-MA DS < TD-MA
Edgin 2010 [25]	IQ matched WS group	WASI	M=17.8 (Range=13-23)	--	*Verbal & Spatial STM* Digit Span Recall Corsi Block Recall	DS<WS DS > WS
Duarte 2011 [183]	MA-matched TD VMA-TD	PPVT	M=12.5 (Range=7-18)	M=4.0	*Verbal & Spatial STM* Digit Span Recall Corsi Span Task	DS < TD-MA & TD-VMA DS < TD-MA; DS ~TD-VMA Also manipulated input & output (verbal vs. visual) of information to be recalled in novel tasks & added spatial support to a visual task. Reported that DS group showed similar performance in pure verbal and verbal-visual tasks. DS group did perform better on visual task with additional spatial support.
*Costanzo 2013* [90]	WS group NVMA-matched TD	Leiter-Revised or SB (1 child matched with SB, all others matched on Leiter-R)	M=14.5 (Range=8-21)	M=6.2	*Verbal & Spatial STM* Digit Span Recall Nonword Repetition Corsi Span Task	DS < WS & TD-NVMA DS < WS & TD- NVMA DS & WS < TD- NVMA; DS ~ WS
Carney 2013 [92]	WS group TD participants with similar MA	SB Abbreviated Battery	M=14.5 (Range=10-21)	M=6.0	*Verbal & Spatial STM* Word List Recall Block Recall	DS~WS~TD DS ~ TD; DS & TD> WS DS Group– Visuospatial > Verbal
Carretti 2013 [94]	VMA-matched TD	PPVT-Revised	M=14.17 (Range=9-17)	M=5.17	*Spatial STM* Spatial-Sequential Test Spatial-Simultaneous Test	DS ~ TD-VMA DS < TD-VMA
Lanfranchi 2014 [93]	MA-matched TD	Logical Operations Test	M=15.66 (SD=3.08)	M=5.75	*Visual STM* Picture Span Recall	DS < TD-MA
Naess 2015 [85]	NVMA-matched TD	WPPSI Block Design	M=6.32 (Range=5-6)	--	*Verbal STM* Sentence Memory Nonword Repetition	DS < TD-NVMA DS < TD- NVMA
Lanfranchi 2015 [184]	MA-matched TD	PPVT-Revised RCM	M=14.17 (Range=9-17)	M=5.17	*Spatial STM* Patterned Spatial Recall Random Spatial Recall	DS < TD-MA DS ~ TD-MA
Loveall 2016 [185]	TD & ID with similar NVMA (measured with the Leiter-R)		M=14.91	M=5.35	*Verbal STM* Nonword repetition	DS < TD-NVMA & ID group
*Danielsson 2016* [91]	MA-matched WS MA-matched TD	SB-abbrev.	M=14.6 (Range=10-21)	M=5.9	*Verbal & Spatial STM* Word List Recall Block Recall	DS ~WS; DS & WS < TD-MA DS >WS; DS ~ TD-MA
Heimann 2016 [61]	TD Language matched ASD	--	M=5.25 (SD=1.03)	M=2.63	*Visual STM* Immediate Elicited Imitation	DS > ASD; DS~TD
**Adult and Older adult studies (18 years and older)**						
Study	Comparison Group	Matching Assessment	CA of DS Group (Yrs)	MA of DS Group	Tasks	Findings
Dalton 1974 [159]	Young & Old ID	--	Young M= 20.9 Intermediate M=41.7	--	*Verbal STM* Digit Span	Young & Intermediate DS < Old ID
Thase 1984 [186]	ID	--	M=37.1 (SD=10.4)	--	*Verbal & Visual STM* Digit Span Delayed Matching to Sample	DS < ID DS < ID
Huang 1987 [109]	CA & MA matched ID	--	M=38.65	--	*Verbal & Visual STM* Digit Span Picture recall	DS ~ ID Adults with DS were more likely to recall a picture in a series of black and white pictures orally if it was in color.
Varnhagen 1987 [64]	CA & IQ matched ID group	SB	M=22.70 (Range=19-25)	M=4.78	*Verbal STM* Auditory Span Recall	DS~ID, but DS group had marginally worse lexical access and did not demonstrate acoustic similarity effect seen in ID group
*Marcell 1988* [84]	ID group VMA-matched TD	PPVT	M=18.12	M=4.98	*Verbal STM* Digit Span Recall (with presentation [auditory vs. visual] and recall [oral vs. manual] differences)	DS< TD-VMA; DS~ID on overall performance collapsed across conditions DS< ID on auditory recall (collapsed across output conditions) TD-VMA & ID: Auditory > Visual Performance DS: Auditory ~ Visual Performance
Marcell 1992 [187]	IQ-matched ID group	SB	M=18.84	--	*Verbal STM* Digit Span Recall Sentence Recall	DS < ID DS < ID
Devenny 1992 [161]	ID < 35 ID > 35	--	<35 M=31.7 >35 M=41.8	--	*Visual STM* Visual Memory Test	>35 DS showed greater decreases in performance at 5 and 10 second delays compared to their performance at 0 second delays than any other participant group
Marcell 1995 [188]	CA & IQ matched ID	SB	M = 18.83 (SD=3.33)	--	*Verbal STM* Sentence Repetition Digit Span	DS < ID on number of sentences repeated and number of words recalled across sentences DS < ID
Devenny 1996 [65]	Young DS (CA 30-39) Middle-Aged DS (CA 40-49) Older DS (CA 50+) ID group with similar IQ & Age	Subtests of WISC-R used to estimate IQ	Young DS M=37 Middle-Aged DS M=45 Older DS M=55		*Visual LTM* Delayed Match to Sample	*Participants tested at 6 time points across 5 years* DS~ID, but DS showed decrements with increased delay Young ~ Middle-Aged ~ Older DS *Note: This study focused on a more mildly impacted subgroup of adults with DS
Vicari 2000 [36]	MA-matched TD	SB	M=21	M=6.5	*Verbal & Visual STM* Word List Recall Word Recognition Picture Recognition	DS<TD-MA DS<TD-MA DS<TD-MA
Natsopoulos 2002 [189]	VMA-matched TD	WPPSI-R Information, Vocabulary & Similarities subtest	M=30.6 (Range=19-39)	--	*Verbal STM* Sentence Repetition	DS < TD-VMA
Brock 2004 [99]	TD group with similar BPVS MA, but not explicitly matched	--	M=18.5 (Range=12-25)	M=7.7	*Verbal STM* Digit Span Recall Word and Nonword Order & Item Recall Memory	DS < TD DS < TD
Kittler 2004 [190]	IQ-Matched ID	WAIS-R or SB	M=44.8	M=6.57 (PPVT-R)	*Verbal STM* Word List Recall	DS < ID on all lists except phonologically similar words; semantic similarity of words reduced DS performance only
Purser 2005 [107]	TD group with similar NVMA	--	M=19.78	M=8.02	*Verbal & Visual STM* Word List Recall Picture Location Recall	DS < TD DS ~ TD
Kittler 2006 [102]	IQ and VMA-matched ID group	WAIS-R and PPVT	M=44.3	M=6.52	*Verbal STM* Word List Recall	DS < ID
Kittler 2008 [103]	MA- matched WS and ID group	--	M=44.7 (SD=7.2)	--	*Verbal & Visual STM* Digit Span Word Span Corsi Span	DS < WS; DS ~ ID DS < WS & ID DS > ID and DS>~WS (p=.057)
Jarrold 2009 [191]	TD group with similar NVMA	--	M=20.82 (Range=14-29)	M=7.33 (PPVT)	*Verbal STM* Serial Recall Serial Recognition	DS < TD DS < TD
*Lee 2010* [98]	VMA-matched TD	PPVT	M=19.18 (Range=11-25)	M=6.95	*Verbal STM* Digit Span Recall	DS < TD-VMA
*Mosse 2011* [192]	VMA-matched TD	BPVS	M=19.5 (Range=9-28)	--	*Verbal STM* Immediate serial recall	DS < TD-VMA
Roch 2012 [122]	Reading comprehension matched TD (CAM)	Standardized test of reading comprehension (administered collectively)	M~18.5 (Range=11-26)	--	*Verbal STM* Sequential word recall Sequential word recall with visual presentation	DS < TD-CAM DS ~ TD-CAM
Michael 2012 [193]	VMA-matched TD	PPVT-4^th^ Edition	M=18.9 (Range=11-32)	--	*Verbal STM* Digit Span Recall Word Span Recall Sentence Memory Nonverbal Digit Span Recall Nonverbal Word Span Recall Nonverbal Spatial Memory (*Nonverbal tasks involved pointing to answers on page)	DS~TD-VMA DS~ TD-VMA DS< TD-VMA DS~ TD-VMA DS~ TD-VMA DS~TD-VMA
Purser 2013 [100]	NVMA- matched TD VMA-matched TD	BPVS- II, RCM	M=19.4 (Range=13-26)	M= 7.9 (BPVS-II)	*Verbal STM* Word List recognition	DS < TD-NVMA & TD-VMA
Smith 2014 [101]	VMA-matched TD	BPVS-II	M=20 (Range=9-30)	--	*Verbal STM* Word Recall Word Order Recall	DS < TD-VMA DS < TD-VMA
*Belacchi 2014* [108]	NVMA and SES matched TD	RCM	M=18.8 (Range=15-29)	MA~5.5	*Verbal & Visual STM* Word Recall Spatial-simultaneous Recall	DS< TD-NVMA DS ~ TD-NVMA
Stavroussi 2016 [104]	CA & VMA ID group	PPVT-R	M=33.92 (Range=26-38)	--	*Verbal STM* Digit Span Recall	DS ~ ID
Reid 2017 [105]	Cornelia de Lange Syndrome group (CdLS)	--	M= 24.38 (SD=5.82)	M=6.29 (BPVS)	*Verbal & Visual STM* Digit Span Corsi Span	DS > CdLS DS > CdLS
**Within DS Group Comparisons of Older Adults**						
Study	Comparison Group	Matching Assessment	CA of DS Group (Yrs)	MA of DS Group	Tasks	Findings
Haxby 1989 [163]	Under 35 DS 35+ DS (4 with dementia)	--	Under 35 M=26 (Range=19-34) 35+ non-demented M=48 (Range=37-54) 35+ demented M=57 (Range= 47-64)	Under 35 M= 6.1 35+ non-demented M=4.3 35+ demented M=2.5 (SB used to measure MA)	*Verbal & Visual STM* Digit Span Recall Block Tapping Span Object Pointing Span	35+ demented < 35+ nondemented & Under 35 35+ demented < Under 35 on Block Tapping Span & Object Pointing Span
Alexander 1997 [66]	Young DS (CA 22-39) Old DS (CA 40-61) without dementia	--	Young DS M=30.1 Old DS M=47.4	Young DS M=5.7 Old DS M=4.7 (SB used to measure MA)	*Verbal & Visual STM* Digit Span Recall Hidden object Memory Recall Block Tapping Span	Old DS ~ Young DS Old DS ~ Young DS Old DS < Young DS
Crayton 1998 [72]	Young DS (CA Under 40) Middle-Aged DS (CA 40-49) Older DS (CA 50+)	--	--	M =4.8 (for whole group on BPVS)	*Visual STM* Matching to Sample	Young DS ~ Middle-Aged DS ~ Older DS

Key: Comparison Groups: *CA* chronological-age, *DS* Down Syndrome, *FXS* Fragile X Syndrome, *ID* Intellectual Disability, *MA* mental-age, *MLD* Moderate Learning Difficulties, *MLU* matched language production, *NVMA* Nonverbal mental-age, *SLI* Specific Language Impairment, *TD* typically-developing, *VMA* Vocabulary (receptive) mental-age, *WS* William’s Syndrome

Tests: *BAS* British Ability Scales, *BPVS* British Picture Vocabulary Scale, *CVLT-C* California Verbal Learning Testing Children’s Version, *DAS* Differential Ability Scales, *EVPT* English Picture Vocabulary Test, *ITPA* Illinois Test of Psycholinguistic Abilities subtest, *K-ABC* Kaufman Assessment Battery for Children, *K-BIT* Kaufman-Brief Intelligence Test, *Leiter* Leiter Performance Intelligence Scales, *PPVT-R* Peabody Picture Vocabulary Test-Revised, *RCM* Raven’s Coloured Matrices, *SB* Stanford Binet Intelligence Scale, *WAIS-R* Wechsler Adult Intelligence Scale-Revised, *WASI* Wechsler Abbreviated Scale of Intelligence, *WISC-R* Wechsler Intelligence Scale for Children- Revised, *WPPSI-R* Wechsler Preschool and Primary Scale of Intelligence-Revised

*Note*: Italicized authors’ names indicate studies included in effect size analyses

^1^These tasks were classified as STM by [83]. Details about the methods of this study made it difficult to ascertain if there was a delay that would result in these being classified as LTM according to our system of task classification

**Table 3:** Studies Examining WM Skills in Individuals with DS Organized by Age Group and Presented Chronologically

Study	Comparison Group	Matching Assessment	DS Group CA	DS Group MA	Tasks	Findings
**School Age and Adolescent studies (6 to 17 years old)**						
Vicari 1995 [118]	NVMA-matched TD ID group	Leiter	M=16.6	M=5.2	*Verbal & Spatial WM* Backwards Digit Span Backwards Corsi Span	DS< TD-NVMA & ID DS< TD-NVMA & ID
*Munir 2000* [58]	MA-matched TD, CA-matched TD, & FXS	BPVS	M= 11.17	M=6.09	*Verbal WM* WISC-III Backward Digit Span	DS > FXS; DS < TD-MA & TD-CA
*Pennington 2003* [9]	MA-matched TD	DAS	M=14 (Range=11-19)	M=4.5	*Verbal & Spatial WM* Counting Span CANTAB Spatial WM	DS ~ TD-MA DS ~ TD-MA
*Lanfranchi 2004* Study 1 [115]	MA-matched TD	Logical Operations Test	M=11.75 (Range=7-16)	M=5.42	*Verbal WM* Backward Word Span Selective Word Recall Dual Request Word Recall	DS < TD-MA DS < TD-MA DS < TD-MA *As cognitive control required for each task increased, the deficit in DS performance increased as well
*Lanfranchi 2004* Study 2 [115]	MA-matched TD	Logical Operations Test	M=14.5 (Range=11-18)	M=4.5	*Visual WM* Pathway Backwards Starting Position Selection Dual Request Selective Task	DS ~ TD-MA DS < TD-MA DS < TD-MA
*Visu-Petra 2007* [120]	MA-matched TD	SB	M=14.4 (Range=8-21)	M=5.75	*Spatial WM* CANTAB Visual Spatial Working Memory (recalling hidden items in sequence)	DS ~ TD-MA on strategyTotal Between Errors: DS> TD- MA 4-Box Between Errors: DS~ TD-MA
*Lanfranchi 2009* [117]	VMA-matched TD Verbal MA-matched TD	PPVT-R WPPSI Verbal Scales	M=13.0 (Range=8-19)	M=4.10	*Verbal & Spatial WM* Selective Span Verbal Double Task Starting Position Selection Visuospatial Double task	DS< TD-Verbal & TD-VMA DS< TD-Verbal & TD-VMA DS~ TD-Verbal & TD-VMA DS< TD-Verbal & TD-VMA
Lanfranchi, 2009 [142]	MA-matched TD	PPVT-R and RCM	M=12.6 (Range=7-17)	M=6.0	*Spatial WM* Selective Position Recall Selective Pathway Recall	DS<TD-MA DS~TD-MA
Edgin 2010 [25]	IQ-matched WS group	WASI	M=17.4 (Range=13-23)	--	*Verbal & Spatial WM* Backwards Digit Span Backward Corsi Span	DS ~ WS DS ~ WS
*Lanfranchi 2010* [116]	MA-matched TD	Logical Operations Test	M=15.1 (Range=11-18)	M=5.75	*Verbal & Spatial WM* Verbal Dual task Visuospatial Dual task	DS< TD-MA DS< TD-MA
Nash 2011 [194]	Comprehension Ability Matched (CAM) TD	BAS-II Single Word Reading Test (RA group) Neale Analysis of Reading Ability II (CAM group)	M=15.5	Reading age=5.2 Rec. Vocab age=7.0	*Verbal WM* Working Memory Test Battery for Children	DS < TD-CAM
*Lanfranchi 2012* [119]	VMA-matched TD	PPVT-Revised	M=13.7 (Range=8-23)	M=5.25	*Verbal & Spatial WM* *Selective Word Recall* *Verbal Dual task* *Verbal/Visuospatial Task* *Selective Pathways Recall* Visuospatial Dual Task Visuospatial/Verbal Task	DS < TD-VMA DS < TD-VMA DS < TD-VMA DS ~ TD-VMA DS < TD-VMA DS < TD-VMA
*Borella 2013* [114]	VMA- matched TD	PPVT- Revised	M=14.6 (Range=10-19)	M=5.58	*Verbal WM* Verbal Dual Task	DS < TD-VMA
*Costanzo 2013* [90]	WS group & NVMA-matched TD	Leiter-Revised (for all but 1 participant who received SB)	M= 14.5 (Range=8-21)	M=6.2	*Verbal & Spatial WM* Backward Digit Span Backward Corsi Span	DS< TD-NVMA: DS<WS DS< TD-NVMA; DS~WS
Carney 2013 [92]	TD participants with similar MA	SB Abbreviated Battery	M=13.6 (Range=10 –18)	M=6.0	*Verbal & Spatial WM* Listening Span Odd One Out *- determining odd figure out of 3 figures, then recalling figure position*	DS < TD DS < TD
Trezise 2014 [121]	CA and NVMA-matched ASD+ID and Nonspecific ID	Wechsler Nonverbal Scale of Ability: Spatial Span MA score	M=14.84 (Range=10-18)	M=8.19	*Verbal & Visual WM* Word Repetition Recognition Line Drawing Recognition	DS < ID; DS~ASD+ID DS < ID; DS~ASD+ID
**Adult and Older adult studies (18 years and older)**						
Numminen 2001 [123]	NV IQ-matched ID group	RCM	M=41.8 (Range=38-48)	M=5.03	*Verbal & Spatial WM* Digit Span Backward Visuospatial Test (recall of patterns)	DS~ID DS~ID
Roch 2012 [122]	Reading comprehension matched TD (CAM)	Standardized test of reading comprehension (administered collectively)	M~18.5 (Range=11-26)	--	*Verbal WM* Backward Word Recall Backward word recall with visual support	DS < TD-CAM DS ~ TD-CAM
*Belacchi 2014* [108]	NVMA and SES matched TD	RCM	M=18.8 (Range=15-29)	MA~5.5	*Verbal & Visual WM* First Word List Recall First Stop Position Recall	DS < TD-NVMA DS = TD-NVMA
Reid 2017 [105]	Cornelia de Lange Snydrome group (CdLS)	--	M= 24.38 (SD=5.82)	M=6.29 (BPVS)	*Verbal & Visual WM* Digits Span Backwards Corsi Backwards	DS~CdLS DS~CdLS

Key: Comparison Groups: *CA* chronological-age, *DS* Down Syndrome, *ID*Intellectual Disability, *MA* mental-age, *NVMA* Nonverbal mental-age, *TD* typically-developing, *VMA* Vocabulary (receptive) mental-age, *WS* William’s Syndrome

Tests: *BAS* British Ability Scales, *DAS* Differential Ability Scales, *Leiter* Leiter Performance Intelligence Scales, *PPVT-R* Peabody Picture Vocabulary Test-Revised, *RCM* Raven’s Coloured Matrices, *SB* Stanford Binet Intelligence Scale, *WASI* Wechsler Abbreviated Scale of Intelligence, *WPPSI* Wechsler Preschool and Primary Scale of Intelligence

*Note*: Italicized authors’ names indicate studies included in effect size analyses

Next, we chose a subset of the 106 studies to gather effect size data to examine the average magnitude of the impairment within each memory domain and modality relative to mental age expectations. The following criteria were imposed to identify tasks from these 106 studies for which effect sizes would be calculated for the current review: (1) the study included a typically developing, mental age-matched comparison group, (2) means and standard deviations were available so that effect sizes could be calculated or effect sizes were reported, and (3) the study provided adequate description of task demands to determine if it should be included in the LTM, STM, or WM sections. Adequate description for LTM tasks included a description of a delay (minutes to days) between memory encoding and retrieval or a description of recall after an extended presentation of stimuli (i.e., includes repeated recall of supraspan item lists in which learning was evaluated over multiple trials). For STM tasks, a description of immediate retrieval of subspan items after encoding was required. Lastly, WM tasks were included if the tasks required immediate retrieval of information and manipulation of the to-be-remembered material. It is important to note that the key distinction between STM and WM studies was the dual task nature of the WM studies, i.e., one not only needs to recall but also must manipulate the material. If a task was described in a study as being a WM task but it did not have a discernible manipulation/dual task component, we included it in the STM table and STM effect size calculations.

When choosing the outcome measure to report on for effect size calculations, if a study presented two outcome variables for one task, we chose the outcome variable that summarized the overall performance best, and consistently chose the same outcome variable for the same task across studies (if possible). Additionally, if the study reported on errors, resulting in a positive effect size indicating worse performance, the sign was reversed (i.e., negative effect size) to maintain consistency and more easily reflect the impaired performance in which negative Cohen’s *d* values reflect poorer performance by the DS group relative to the MA group.

Exclusionary criteria for evaluating effect sizes were as follows: (1) non-parametric statistical analyses were used in the original paper and/or the authors of the paper noted that the distribution of the data for a particular task was non-normal, suggesting that the mean may not be the most suitable measure of central tendency, (2) the manuscript described performance of subgroups of individuals with DS rather than the group as a whole (e.g., studies where good vs. poor readers with DS were described), (3) tasks were used to examine factors that influence memory impairments in DS rather than quantify the extent of impairment relative to controls, and (4) the modality of stimuli presentation differed from the modality being evaluated (i.e., visual presentation for verbal recall). Further, for the STM domain, effect sizes were not calculated for nonword repetition or sentence memory tasks due to the concern that STM difficulties might be overestimated and due to prominent articulation and syntactic processing deficits in DS (see [50] for a review), respectively. Similarly, effect sizes were not calculated for studies employing story memory tasks, given the added syntactic processing demands of such tasks.

Decision to include a study in the domain in which it was placed was established by consensus between the first and second authors. For transparency about the demands of the tasks that fell into the different domains—LTM, STM, and WM—and for effect size calculations, we have provided detailed task descriptions in a supplementary table (Additional file 1: Table S1). Using the established criteria, 26 of the 106 studies contributed data for effect size calculation; from these studies, 71 effect sizes were calculated. Of these 71 effect sizes, 38 (53%) were derived from studies with an overall MA-matched TD group, 9 (13%) were matched on nonverbal MA, and 24 (34%) were matched on verbal MA (as measured in nearly all cases by a receptive vocabulary task). We then plotted effect size estimates as a function of mean chronological age of the DS group for the LTM, STM, and WM studies reviewed in order to summarize existing data visually. These figures include DS and TD comparisons only (i.e., not DS and ID comparisons). Some studies used multiple tasks, and thus, the tasks are plotted separately (i.e., a study’s findings can be found in multiple figures if tasks tapped different memory domains; also, if multiple measures of the same memory domain were included, these are plotted separately in the same figure).

## Long-term memory

LTM refers to the process of storing information that can be retrieved for use in minutes, hours, or years later. It involves three stages: encoding (i.e., extracting distinct factors to form a memory), storage (i.e., maintenance of memories), and retrieval (i.e., obtaining information from storage), and is thought to call upon a complex network of neural structures, including the hippocampus, the perirhinal cortex, and the parahippocampal cortex [51]. LTM is often assessed using tasks in which an individual is introduced to a novel set of stimuli and then asked to recall that information over several trials and/or following a delay (e.g., 20 to 30 min). Examples of standardized verbal and visual LTM tasks include the California Verbal Learning Test (CVLT) and the Rey Complex Figure Test (RCFT), respectively [52, 53]. The CVLT is a list-learning task in which participants must recall supraspan lists of words that are repeated over several trials and following a delay. The RCFT involves recalling the configuration of a complex figure both immediately and following a delay. These two types of tasks tend to be used with school-age and older participants with DS to evaluate LTM. Examples of tasks used with children prior to the school-age years (before the age of 5) tend to involve delayed imitation (e.g., learning a three-step action sequence and recalling after a delay) and object location memory (e.g., recalling the location of a toy after a delay). LTM abilities across the lifespan in DS are described in the following sections. In addition, Table 1 summarizes the existing research literature in greater detail.

**Fig. 1:**
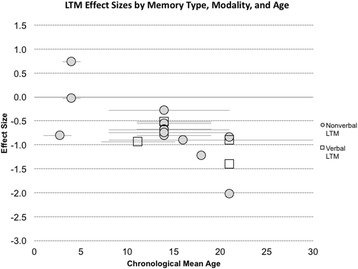
LTM effect sizes from past studies, divided by presentation modality. Effect sizes calculated using Cohen’s *d* ((DS group mean–control group mean)/pooled standard deviation) for studies that reported effect size data. An effect size of zero indicates equivalent performance between the DS and control group. A total of 16 effect sizes were calculated for LTM. All control groups were typically developing children matched on overall mental age abilities. Lower scores indicate worse impairment. Although a greater number of LTM studies exist, the data provided in additional studies precluded the calculation of effect sizes or did not meet our criteria for inclusion. Descriptions of studies included in this figure can be found in Table 1 and Additional file 1: Table S1 (for detailed task descriptions)

### Preschool studies (≤ 5 years old)

No studies of which we are aware examine verbal LTM abilities in preschool children with DS; consequently, our knowledge of LTM in the verbal modality does not begin until school age. In contrast, there are a few studies that have examined nonverbal LTM abilities in the preschool age, but findings are mixed. Roberts and Richmond (2015) found preschool-age children with DS performed comparably to their MA-matched peers on object location recall and deferred imitation tasks after a 24-h delay [35]. Consequently, these researchers concluded that LTM impairments (in excess of MA) did not begin until after the early childhood years in DS. In contrast, Milojevich and Lukowski (2016) reported impaired performance on recall of sequenced information by preschool-age children with DS compared to MA-matched peers after a 1-month delay. Therefore, the researchers contended that LTM impairments (in excess of MA) are present in preschool children with DS [54]. Thus, the limited data from preschool suggest that although children with DS may have MA expected nonverbal LTM performance after a shorter delay (24-h), their performance appears worse than MA expectations after a month-long delay. Clearly, more research is needed to clarify LTM abilities in the early developmental years of DS. Such research could help clinicians identify target ages to provide memory interventions, perhaps prior to the onset of significant deviations from mental or even chronological age expectations.

### School age and adolescent studies (6 to 17 years old)

Within LTM research, there has been a consistent finding of significant verbal LTM impairments among adolescents with DS. With the exception of one study [55], DS groups perform significantly worse than MA-matched TD peers on list-learning tasks with a delay [9, 56, 57]. Additionally, Nichols [57] found adolescents with DS had significantly more intrusive responses and impaired discrimination abilities on list recall compared to MA-matched controls.

In comparison to children with ID, several studies have reported that teens with DS performed significantly worse on verbal LTM tasks [56, 58, 59]. However, when compared to children with Williams syndrome (WS), teens with DS have been reported to have equivalent performance on verbal list-learning long-delay recalls (e.g., CVLT-Children’s Version) [57] and better performance on a word list learning task [25]. Thus, although research indicates adolescents with DS perform below MA-matched typically developing controls on verbal LTM assessments, additional research is needed to clarify whether this deficit is comparable or less significant than that found in other ID groups.

Similar to their verbal LTM abilities, most studies report that adolescents with DS demonstrate impaired performance on nonverbal LTM tasks in comparison to TD children matched on MA (e.g., [9, 56, 60], but see [61, 62]). Specifically, teens with DS exhibit impaired performance on visual associative memory tasks, pattern recognition, and spatial LTM tasks (e.g., maze location and spatial location recall tasks; [9]) compared with MA-matched children.

However, teens with DS largely have similar or stronger performance when compared to groups with mixed ID or WS on visual LTM tasks [55, 56]. In addition, adolescents with DS have demonstrated significantly greater performance than children with WS on spatial LTM tasks [25]. Thus, there appears to be consistent evidence that adolescents with DS perform comparably or better than other ID groups, yet worse than MA-matched TD comparison groups on nonverbal LTM tasks in the majority of research studies.

### Adult and older adult studies (18 years and older)

Adults with DS continue to perform below MA expectations on LTM tasks. The very limited data available suggest that on verbal LTM tasks, adults with DS perform worse than MA-matched typically developing controls [36]. Similarly, several studies that have compared adults with DS to ID groups have reported lower performance [63–65], which includes research that has shown that adults with DS have significantly longer response latencies on verbal memory tasks [64] and lower levels of improvement across repeated testing [65] relative to other ID groups. Furthermore, unlike other ID groups, performance on word list learning tasks is inversely related to age in adults with DS [63, 66]. Additionally, younger adults with DS show improvement with repeated testing of verbal LTM list-learning tasks (e.g., selective reminding task), while older adults actually exhibit a small decrease in performance across testing [65].

Research has also shown that adults with DS perform significantly worse than MA-matched TD controls on nonverbal LTM tasks [36, 67–69]. Research comparing nonverbal LTM in those with DS to other ID groups reveals mixed findings, with some studies reporting similar performance on some tasks ([70, 71]) and greater impairment on others [69, 70]. Research within the DS population has demonstrated that nonverbal LTM memory begins to decline in young adulthood [72, 73]. In particular, performance on tasks with higher cognitive load (i.e., increased number of stimuli) significantly declines with age in adulthood [74, 75].

The majority of adult research comparing LTM abilities (both verbal and nonverbal) of DS groups to TD groups focuses on individuals with an average age in the 30s. Many studies examining memory abilities of older adults do exist. However, these studies typically compare adults with DS at different age points, rather than comparing to mental age-matched TD participants. Consequently, the field lacks research examining older adults with DS compared to those with typical development.

## LTM across development in DS

To summarize the existing literature in DS across development visually, we calculated effect sizes using Cohen’s *d* [76] (i.e., DS group mean–MA-matched TD control group mean/pooled standard deviation) for published studies that fit our inclusion/exclusion criteria (delineated in the “Literature review and analytic method” section). These studies are in italics print in Table 1. We have summarized the effect sizes of findings from existing LTM studies of DS as a function of the mean chronological age of the DS group and task modality in Fig. 1. In addition, in this figure, we have noted the method by which the DS group was matched to the typically developing group (verbal, nonverbal, or overall MA) and included details about the assessment tool used to match DS and TD participants in Table 1. Additional file 1: Table S1 provides greater details about the tasks included in Fig. 1.

First, collapsing across studies ignoring age, we find the mean effect size for the LTM domain is medium to large (*d* = −.73 overall). The effect size for verbal LTM (*d* = −0.94) is large, while the effect size for nonverbal LTM is medium (*d* = −.68). Thus, it is clear that LTM abilities deviate from mental age expectations in DS. Moreover, impairments are evident (in most studies) from an early age and persist across development. There is a suggestion in the data of greater nonverbal LTM impairments relative to mental age expectations later in development. However, given the differences in the tasks used across development, different mental age matching strategies, and the lack of longitudinal research, this observation is made very tentatively.

## Short-term memory

STM refers to a limited capacity, immediate memory system in which small amounts of information can be actively upheld and preserved for a matter of seconds. With regard to its neural correlates, STM encoding and retrieval involve a network of regions, including the frontal lobes, inferior portions of the parietal lobe, hippocampus, and superior portions of the temporal lobe [77–80]. However, neuroimaging data suggest that somewhat separate cortical networks underlie verbal vs. visual STM performance. For example, phonological storage is thought to involve the left posterior parietal cortex while rehearsal is thought to involve the left premotor cortex, Broca’s area, and supplementary motor cortex (for a review, see [81]). In contrast, visual storage is thought to rely upon the right anterior occipital cortex, while rehearsal is thought to involve the right posterior parietal and premotor cortex (for a review, see [81]).

**Fig. 2:**
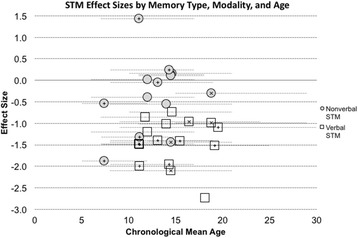
STM effect sizes from past studies, divided by presentation modality. Effect sizes calculated using Cohen’s *d* ((DS group mean − control group mean)/pooled standard deviation) for studies that reported effect size data. An effect size of zero indicates equivalent performance between the DS and control group. A total of 30 effect sizes were calculated for STM. Eleven effect sides were based on control groups of typically developing children matched on overall mental age abilities, 14 effect sizes were based on controls matched on verbal MA (using a vocabulary test), and five were matched on nonverbal MA. Lower scores indicate worse impairment. Although a greater number of STM studies exist, the data provided in additional studies precluded the calculation of effect sizes or did not meet our criteria for inclusion. Studies included in the effect size analyses can be found in Table 2 and Additional file 1: Table S1 (for detailed task descriptions)

To assess STM, visual or verbal span tasks are typically administered, such as Corsi Span or Digit Span, respectively, in which individuals must recall a sequence of blocks (visual) or digits (verbal) verbatim [82]. Other examples of STM tasks include immediate subspan word list recall (verbal), pattern recall (visual), and picture location recall (visual). We summarize the existing literature by age group in the sections that follow. In addition, we provide details about studies of STM in DS in Table 2, and age-effect size relations are displayed in Fig. 2.

### Preschool studies (3 to 5 years old)

Very limited research exists that examines STM abilities in very young children with DS. Existing research suggests impairment relative to youth with FXS [83]. However, it is clear that more STM research is needed during this developmental period. With that, we turn to studies during the school-age and adolescent period.

### School age and adolescent studies (6 to 17 years old)

During childhood and adolescence, the vast majority of research suggests that individuals with DS perform worse than MA-matched peers on verbal STM tasks such as digit span and immediate word list recall (e.g., [9, 49, 84]). Additionally, research suggests children and adolescents with DS make smaller gains on verbal STM tasks across development in comparison to MA-matched TD children, and this deficit becomes worse with age [85–87].

The majority of research comparing DS to ID groups has found adolescents with DS show greater verbal STM impairments than other ID groups, as well as children with speech language impairments, and children with focal brain lesions (e.g., [84, 88–90]). However, research findings are mixed, with several researchers reporting comparable verbal STM skills to those with other forms of ID (e.g., [58, 91]).

In comparison to their verbal STM abilities, adolescents with DS have relatively stronger nonverbal STM skills [92]. However, findings regarding whether these skills are below mental age expectations when compared to typically developing youth and youth with other forms of ID are variable. In comparison to TD youth, most studies suggest comparable or poorer performance on nonverbal STM tasks (e.g., [93–95]). In comparison to other ID groups, most studies report comparable or stronger performance (e.g., [25, 58, 96, 97]). Therefore, while research has demonstrated nonverbal STM skills are a relative strength compared with verbal STM abilities within DS, findings are mixed with regard to whether these skills differ from those observed in TD or ID peers matched on mental age during this time period.

### Adult and older adult studies (18 years and older)

As individuals with DS age, research has shown STM abilities remain impaired. Compared to MA-matched controls, including both verbal-matched and nonverbal-matched controls, young adults with DS are significantly impaired on verbal STM tasks such as digit and word recall tasks [98–100]. Additionally, DS groups perform worse in comparison to MA-matched TD controls on tests requiring recall of a missing item (item memory) or an entire list (serial memory) [101].

Research comparing adults with DS to those with other forms of ID on verbal STM tasks is mixed. While the majority of studies demonstrate greater impairments relative to other ID groups (e.g., [102, 103]), several studies report similar levels of impairment ([64, 104]), and a recent study that compared adults with DS to adults with Cornelia de Lange syndrome revealed stronger performance in the DS group [105].

Although research has suggested that nonverbal STM abilities are relatively stronger than verbal STM skills in DS [106], few studies have compared nonverbal STM in DS to MA-matched controls or other ID groups in adulthood. The limited data suggest that adults with DS perform comparably to TD comparison subjects with similar mental ages (e.g., [107, 108], but see [36]), and other adults with ID (e.g., [65, 109]), suggesting that nonverbal STM impairments are relatively in line with mental age during adulthood.

## STM across development in DS

We have summarized the effect size data from past studies in Fig. 2, which depicts STM effect sizes relative to MA-matched comparison subjects using Cohen’s *d* organized by age and task modality. (See Table 2 and Additional file 1: Table S1 for greater details about the studies and tasks included in Fig. 2.) The average Cohen’s *d* value collapsed across verbal and nonverbal STM studies was − 0.91. This large effect was driven by studies of verbal STM for which the effect size was large (− 1.40), suggesting significant impairments in excess of overall cognitive limitations. In contrast, the small effect size for nonverbal STM of − 0.32 suggests that nonverbal abilities only slightly deviate from mental age expectations.

Looking across the ages studied, there is consistent evidence for verbal STM impairment in DS relative to MA-matched comparison groups beginning in school age and into adulthood. However, it is important to note that limited data are available in early childhood. Turning to nonverbal STM, effect size data are limited to a very restricted age range, with the preponderance of studies between the ages of 11 and 14. These data suggest nonverbal STM performance is somewhat weaker than MA-matched peers, although this is based primarily on inconsistent findings across studies with adolescents.

## Working memory

WM, or “working with memory” [110], refers to the ability to maintain and manipulate information for a brief period of time. Similar to STM, WM involves temporary storage of limited amounts of information, but it also requires maintenance and attention while simultaneously processing information, avoiding distraction, and/or engaging in cognitive shifting [111, 112]. As reviewed by Wager and colleagues [113], WM activates a wide network of neural regions, including numerous frontal and parietal regions. These regions appear to differ based on the nature of the tasks. For example, in tasks requiring updating and sequencing information, the dorsolateral prefrontal cortex and the superior frontal sulcus are involved, while manipulation tasks require the ventral and anterior prefrontal cortex.

Assessments of WM are often similar to STM, with additional components requiring increased attention and/or manipulation of the to-be-remembered stimuli. Verbal WM tasks include digit span backward, backward word span recall, and selective word recall (e.g., hearing multiple lists of words and recalling the first word from each list after the presentation of all of the lists). Nonverbal WM tasks include spatial span backward or tasks that require recall of the first step in a series of steps.

**Fig. 3:**
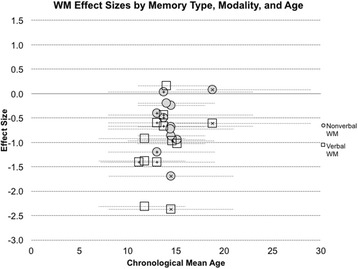
WM effect sizes from past studies, divided by presentation modality. Effect sizes calculated using Cohen’s *d* ((DS group mean − control group mean)/pooled standard deviation) for studies that reported effect size data. An effect size of zero indicates equivalent performance between the DS and control group. A total of 25 effect sizes were calculated for WM. Control groups were typically developing children matched on overall mental age abilities, except for 14 effect sizes used (ten effect sizes were based on control groups matched on verbal MA (using a vocabulary test), and four effect sizes used controls matched on nonverbal MA). Lower scores indicate worse impairment. Although a greater number of WM studies exist, the data provided in additional studies precluded the calculation of effect sizes or did not meet our criteria for inclusion. Studies included in the effect size analyses can be found in Table 3 and Additional file 1: Table S1 (for detailed task descriptions)

Overall, research on WM abilities in DS remains limited across the lifespan. Very few studies have examined these abilities in early childhood or in adulthood. However, the majority of research studies report that adolescents and young adults with DS perform below MA expectations on both verbal and nonverbal WM tasks. Existing studies are summarized in detail in Table 3 and age-effect size relations are illustrated in Fig. 3.

### Preschool studies (3 to 5 years old)

No studies of which we are aware have examined WM abilities using direct cognitive assessment in young children with DS. This is likely due to the complexity of such tasks and difficulties adapting WM tasks to be understandable to very young children. Thus, we turn our attention to studies of school-age children and adolescents.

### School age and adolescent studies (6 to 17 years old)

Similar to performance on verbal STM tasks, children and adolescents with DS are significantly impaired on verbal WM tasks. With few exceptions (e.g., [9]), youth with DS perform significantly worse on verbal WM tasks compared to MA-matched children and this deficit increases as the task difficulty increases (e.g., [92, 114–116]). Furthermore, even when matching children on vocabulary abilities or reading comprehension abilities, children with DS consistently perform worse than TD children on verbal WM tasks [117]. In contrast to the consistent findings relative to MA-matched peers, the limited studies available have provided conflicting results when comparing performance of groups with DS to other ID groups [25, 90, 118].

Analogous to verbal WM findings, many studies suggest that children and adolescents with DS have significant nonverbal WM impairments compared with MA-matched peers [90, 92, 115–117, 119]. However, findings are mixed with several studies noting similar performance to MA-matched TD participants [9, 119, 120]. In comparison to other ID groups, findings are also mixed; some studies (e.g., [25]) found children with DS have comparable nonverbal WM abilities, while others have reported greater impairments compared to other ID groups [90, 118, 121]. Therefore, further research is needed to determine if nonverbal WM is a deficit beyond MA expectations and if this deficit is unique to DS.

### Adult and older adult studies in DS

Research regarding WM abilities in adults with DS is very limited. The available data indicate that adults with DS perform worse than MA-matched TD individuals on verbal WM tasks [108, 122], yet do not significantly differ from other ID groups [123]. With regard to nonverbal WM abilities in adults with DS relative to TD comparison groups, only one study has examined this and reported comparable performance [108]. In comparison to other ID groups, the very limited data suggests comparable performance as well [105, 123]. However, research examining aging in DS (without a TD comparison group) suggests declining executive function abilities beginning in middle adulthood and that these abilities are one of the first cognitive skills to deteriorate in the early stages of dementia [124–126]. Because WM is thought to be a form of executive functioning, studies that examine WM from adolescence into young and middle adulthood may help to identify precursors to dementia in those individuals with DS who will go on to develop it.

## WM across development in DS

The effect size findings of past studies are summarized in Fig. 3. (See Table 3 and Additional file 1: Table S1 for greater details about the studies and tasks included in Fig. 3). As can be seen, the majority of WM studies have been conducted with adolescents. Thus, our ability to describe WM abilities across the lifespan in DS is quite limited. Of the existing studies, the average Cohen’s *d* value collapsed across verbal and nonverbal WM studies is − 0.81, suggesting a large effect. The mean effect sizes for verbal WM and nonverbal WM considered individually are medium to large (verbal WM Cohen’s *d* = − 1.0; nonverbal WM Cohen’s *d* = − 0.58). Thus, there is a suggestion that WM impairments are significantly greater than global cognitive impairments in DS in adolescence, but more research is needed earlier and later in development.

## Discussion

In this review, we summarized the literature on memory impairments in DS from a developmental perspective by grouping studies into three developmental periods: preschool, school-age and adolescence, and adulthood. We presented findings on LTM, STM, and WM (distinguishing between the verbal and nonverbal modalities) and examined the degree of impairment (measured using Cohen’s *d*) relative to mental age-matched TD controls across studies in each domain. In addition, we summarized the results of studies in which DS performance was compared to that of another ID group to evaluate the degree to which the reported impairment appeared to be specifically associated with DS or rather a more general correlate of intellectual disability. These findings are summarized in the sections that follow.

With only a few exceptions [35, 55, 61, 62], our review of LTM abilities suggests impairment across development in excess of overall cognitive impairment. As it is difficult to study verbal LTM in young children, the existing literature on DS identifies impairment in excess of overall cognitive abilities (i.e., below mental age) by adolescence and continuing into adulthood. Turning to nonverbal LTM, there is evidence for impairments in excess of global learning difficulties beginning during the preschool period (e.g., [36, 54, 69], but see [35]) and continuing through the school-age years and into adulthood (see Table 1 and Fig. 1). Additionally, a review of findings across development suggests that LTM impairments may be somewhat greater later in life. However, this observation is very tentative, as the appropriate research studies have not been conducted to truly describe changes in LTM across the lifespan. Thus, further research should examine the relation between age and nonverbal LTM impairment through longitudinal investigations that track the same participants over the course of development and, importantly, compare performance not only to mental age expectations but also to chronological age expectations. This latter comparison will provide a true evaluation of trajectory differences between individuals with DS and typically developing peers, thus providing a better estimate of how LTM skills in DS deviate from age expectations over time.

Our review provided support for the presence of a significant verbal STM impairment compared to MA-matched typically developing groups. This impairment was evident from the school-age years and persisted into adulthood. In contrast, research findings on nonverbal STM abilities (particularly during the school age/adolescent period) are mixed. Yet, it is important to note that our review of effect sizes for nonverbal STM suggests a small effect (− .32) relative to TD controls matched on mental age overall. This effect is substantially smaller than that seen for verbal STM (− 1.4), suggesting that STM is not universally below mental age expectations in DS. Rather, impairments are much more pronounced when verbal STM is considered (see Table 2 and Fig. 2).

Research on WM abilities in DS across the lifespan is extremely limited. This is in part due to the fact that the majority of research examining limited capacity memory systems used traditional STM tests, rather than WM tests (i.e., tasks that require short-term retention and manipulation of the to-be-remembered material). Nonetheless, the preponderance of studies report that verbal WM abilities are below MA expectations from adolescence into adulthood. Studies evaluating nonverbal WM present varying results relative to mental age-matched TD groups. Unfortunately, we are limited in what we can say about WM (verbal and nonverbal) abilities in adulthood, as studies are sparse. Due to the scarcity of WM research across different developmental periods and the potential impact it could have for clinical care and interventions in DS, future research is needed to provide a better understanding of WM abilities across the lifespan in this population.

In this review, we also identified studies that compared DS performance to that of peers with ID to evaluate whether the impairment in DS is greater than that seen in groups with another form of ID, or if it may represent a more general characteristic of ID. Of the research examining memory abilities in ID groups, there is the largest number of studies examining STM. Within these studies, WS was the most frequently compared group to DS. There is a long-standing recognition of a so-called double dissociation between verbal and nonverbal STM abilities for these groups, such that those with WS are reported to have stronger verbal STM skills, while those with DS are reported to have stronger nonverbal STM skills (see Table 2). However, a few more recent studies reported no differences between these groups on verbal STM ([91, 92]). Studies comparing verbal STM abilities of those with DS to other ID groups are mixed, but the majority of studies suggest greater verbal STM deficits in DS than in other ID groups [58, 64, 84, 88, 89, 91, 102, 127]. Lastly, findings for nonverbal STM as well as both verbal and nonverbal LTM and WM are also mixed when comparing the performance of those with DS to other ID groups (see Tables 1, 2, and 3).

Thus, based on our review of the literature, there appears to be a lack of conclusive evidence regarding whether STM, LTM, and WM impairments in DS differ in degree from those in the other ID groups. Of all of the domains and modalities reviewed, there is the greatest evidence that verbal STM impairments are weaker than in other ID groups. However, given that findings are mixed, strong conclusions about the specificity of this deficit to DS cannot be made. Such mixed findings across LTM, STM, and WM suggest that impairments in memory may characterize ID more generally. However, given the limited number of studies for certain domains, particularly LTM and WM (especially in adulthood), it appears that future research is needed to answer this question. One area that could benefit from greater research in the future is the study of WM in adulthood (both comparing DS to ID and TD groups). Such research may inform studies examining the development of AD in adults with DS, given that research suggests that executive function (which includes working memory as a subdomain) impairments are among the first to emerge in those with DS who develop AD [124–126].

Having summarized the existing behavioral literature on memory impairments in DS, we now turn to a brief review of the neural correlates of memory and how these can be conceptualized within the context of the DS neural phenotype. Given the profound impairments in explicit memory systems observed in individuals with DS, the medial temporal lobes and the hippocampal formation in particular have been the focus of several neuroimaging investigations in DS. Consistent with conclusions drawn from behavioral memory studies, imaging studies have documented reduced volume in the hippocampi and connectivity disturbances in the limbic system of individuals with DS across the lifespan [128–131]. Turning to STM and WM, studies of these skills in those with typical development and those with acquired lesions have identified the central roles of the parietal and frontal lobes for the completion of such tasks [132, 133]. Consistent with these findings, neuroimaging investigations have revealed that in addition to reduced whole-brain volume, children and adults with DS have reduced frontal gray matter volume and parietal white matter volume, as well as lower levels of activation in the parietal lobes compared to TD peers [128, 134–136]. Moreover, youth with DS have increased cortical thickness in the frontal and parietal lobes, suggesting possibly less “mature” cortex in these brain regions [137]. Furthermore, because STM and WM processes require interaction between cortical regions and the hippocampus ([77–80]), behavioral data suggest impairment in connectivity among neural regions in DS. Past research has shown unique patterns of neural activity in children with DS and, in particular, under-connectivity between distant neural regions in those with DS compared to TD controls [6, 138].

Lastly, as previously stated, AD neuropathology results in neurodegeneration of the brain, particularly in the medial temporal and frontal lobes. Imaging studies of the DS population have revealed age-related atrophy in the frontal and medial temporal lobes, particularly in the hippocampus, similar to the beginning stages of AD in the TD population ([139, 140]). Furthermore, studies have shown hippocampal atrophy in adults with DS is correlated with their performance on memory measures ([141]). However, neuroimaging data of the DS population has shown atypical volume, as well as atypical connectivity, in the frontal lobe and medial temporal region, prior to the onset of AD [128–131]. Consequently, it appears that AD neuropathology further alters already atypical neural structures in DS.

### Limitations and future directions

The current review is not without limitations. We will note these here and focus on a few of these limitations to draw attention to avenues for future research. First, in the current review, we have attempted to examine the degree to which memory impairments in DS deviate from mental age expectations at different age points. However, a more effective way to examine developmental trajectories of memory skills is to compare DS performance to chronological age expectations. This limitation in our review is driven by the state of the literature, as the vast majority of studies of individuals with DS compare performance to younger, typically developing individuals based on mental age. In most instances, it would be a foregone conclusion that individuals with DS would perform more poorly than those of the same chronological age without ID. Thus, the desire in studies using MA-matched comparison groups is to demonstrate that challenges in a particular memory domain are in excess of overall cognitive limitations. However, the inherent difficulty with this approach is that our descriptions of cognitive abilities in DS (and other forms of ID) are relative to overall mental abilities, which by definition are atypical in this population. If the true desire is to demonstrate the degree of impairment and ultimately track this over time, then a chronological age-matched comparison group would be more appropriate. However, this leads us to another difficulty that is likely to be contributing to the bias in the field, namely, limitations in current assessment tools.

The availability of current instruments to evaluate memory or other domains of neuropsychological functioning is greatly limited by the developmental level of the targeted participants. Often separate tests are developed for pediatric and adult populations, likely due to difficulties designing tasks that would be easy enough for a child to complete but challenging enough for an adult to complete. Thus, researchers are left with the task of identifying assessment tools that will be appropriately challenging for all participants in their study. However, to truly understand how DS and other ID groups’ performance on different neuropsychological domains vary as a function of chronological age, better assessment tools are needed. Thus, future research should focus on developing such assessments. While there are a few instruments available now that capture individual differences across a wide age range (e.g., NIH toolbox, some intelligence tests), more assessment tools are needed to measure memory from infancy to adulthood.

Another potential limitation of the current review is that some tasks (especially experimental ones) that were classified as belonging to one of the three memory domains (i.e., LTM, STM, WM) could be deemed by another researcher as belonging to a different domain. We encountered this on a few occasions when distinguishing between STM and WM studies. Specifically, some researchers (e.g., [142]) classified tasks as WM tasks (with low cognitive load) that we classified as STM tasks using our operational definitions of the two constructs. To increase transparency about our classification decisions, we (a) provided detailed definitions of the distinctions between LTM, STM, and WM in the methods, (b) gave examples of the types of tasks that fall into these different memory domains in each section of the review, and (c) provided detailed descriptions of task demands for the LTM, STM, and WM tasks included in our effect size analyses in Additional file 1: Table S1.

While we recognize this limitation in the current review, it is interesting to note that theories of memory fragmentation are evolving and tending to rely less on the temporal dimension of memory domain distinctions (e.g., the difference between STM and LTM) and instead focus more on the content to be remembered. For example, based on both human and animal research on the neural systems underlying different aspects of memory, Nadel and Hardt [48] argue that a representational view of memory, rather than one that emphasizes the temporal aspects of memory recall, may more accurately capture how the brain stores memories. This more nuanced view of memory has been applied in recent research studies of DS (e.g., [143]) and has been fruitful in identifying congruence between performance on different memory tasks by humans with DS and animal models of DS. Thus, while the state of the existing literature on DS necessitated the use of these classical memory distinctions, evolving theories of the embodiment of memory in the brain suggest that new constructs and distinctions are likely to result in a revised description of the nature of memory impairments in DS.

Fourth, as is apparent from a review of the tables and figures in this paper, the bulk of research on memory abilities in DS has been conducted with teens and young adults. As a result, we know very little about the development of these skills in preschool and early childhood and in later adulthood. Currently available cognitive assessments used to examine these skills are not adequate to examine young children with DS. Consequently, without adequate assessments, research has neglected to examine these memory domains in younger children. Given that DS is associated with precocious-onset Alzheimer’s disease, which involves declining LTM skills [18] and executive functions [125, 126], more research is needed early and later in life in order to document the typical trajectory of these skills in DS. Identifying the typical trajectory of memory abilities could support the identification of developmental periods during which the application of interventions would be most beneficial. Additionally, such research may aid efforts to identify Alzheimer’s disease symptoms sooner so that earlier interventions may be implemented for those affected.

Fifth, in addition to the need to expand our knowledge of the lifespan trajectories of different domains of memory function, there is also a need for greater research on the relationships between memory and real-world outcomes, such as IQ, academic success, and adaptive functioning. Past research has suggested both STM and LTM abilities are associated with intelligence scores (i.e., STM *r* = .84, LTM *r* = .52; [25]), adaptive behavior (i.e., STM *r* = .0.39, LTM *r* = .28; [25]), and language abilities (i.e., combined STM and LTM scores *r* = .72; [9]) in DS. However, further research, particularly using a longitudinal design, could help pinpoint the cognitive underpinnings of the complex learning deficits in DS and thus inform educational strategies for these individuals.

Furthermore, although research has consistently documented adaptive function deficits in individuals with DS [5, 6, 144], research examining the relation between memory and adaptive function remains very limited. The two studies examining this relationship found that LTM performance is predictive of adaptive function in teens with DS, after accounting for age [9, 25]. However, no studies of which we are aware examine this correlation in childhood or adulthood or utilize a longitudinal design to evaluate this relation over time. If concurrent and predictive associations do exist, interventions that target memory (pharmaceutical or behavioral) could aid adaptive function and, consequently, improve quality of life and independence skills across the lifespan. Therefore, gaining a better understanding of the relation between memory and adaptive function in the DS population is vital due to the importance of adaptive function skills for independence [145].

Moreover, more research on memory interventions is needed across development. With regard to behavioral interventions, research examining memory training effects for those with DS has primarily focused on immediate recall or recognition skills (i.e., STM). For example, verbal STM abilities have been shown to improve through memory training that includes routine practice of STM tasks, and practice of immediate rehearsal techniques ([146, 147]). Encouragingly, the limited research available has demonstrated that improvements in verbal STM are associated with language comprehension performance, suggesting verbal STM training may also improve language abilities in this group [146]. Complementing intervention studies targeting verbal STM, research suggests improvements in visual STM performance in youth with DS after routine memory training ([148, 149]). Additionally, children who completed memory training demonstrated transfer effects (to other, untrained STM tasks), suggesting that this training can result in generalization beyond trained tasks to other types of tasks requiring STM skills ([148, 149]). Future research should continue to focus on the utility of STM interventions to shrink the gap between STM abilities and overall cognitive abilities and more importantly improve real-world outcomes in language and reading abilities that have been linked to this memory domain.

In addition to memory training, pharmacotherapy in the DS population has been an area of focus in recent years. Preclinical research on the Ts65Dn mouse, the best characterized murine model of DS, created a strong basis of understanding for clinical research. Specifically, studies of the Ts65Dn mouse have shown that interventions targeting GABA and NMDA receptors can improve memory functioning, and pharmaceutical interventions using neuroprotective agents and antioxidants can target neurodegeneration (i.e., AD in DS; for review, see [150]). However, clinical studies on individuals with DS have found mixed effects for the pharmaceutical interventions successfully used for the Ts65Dn mouse model. For example, clinical studies examining antioxidant therapy have failed to demonstrate cognitive improvement in both children and adults with DS ([151, 152]). Additionally, donepezil, thought to maintain levels of acetylcholine, which influence memory and language abilities, has failed to improve cognitive measures in individuals with DS ([153]). A recent clinical trial has focused on the use of EGCG (epigallocatechin-3-gallate) to improve memory functioning ([154]). EGCG is an inhibitor of DYRK1A, a triplicated gene thought to influence the expression of ID in DS. This study found improvement in visual and verbal STM after 12 months of EGCG therapy. Lastly, memantine, an NMDA antagonist, has produced mixed findings among clinical trials. Boada ([155]) found adults with DS (ages 18 to 32) had proved verbal (CVLT) and nonverbal (paired associates learning) LTM performance compared to a placebo group. However, Hanney [156] found that memantine therapy had no effect for individuals with DS over the age of 40, suggesting that the AD neuropathology may be too far progressed at this age point for memantine to improve functioning. Although there is still much research to be done in this field, at least a few of the published early clinical studies demonstrate the potential efficacy of pharmaceutical interventions, and the need for earlier interventions before AD onset.

Lastly, research on memory abilities in DS could be supplemented by examining implicit memory and the neural systems underlying these abilities, including the basal ganglia and cortico-striatal networks (for a review, see [31]). Further exploration of this memory domain and research findings that identify similar basal ganglia volumes in adults with DS and chronological age-matched controls [135] could provide insights into neurobiological underpinnings of this relative memory strength and possibly identify compensatory targets for intervention to improve learning and memory in DS.

## Conclusion

In conclusion, while behavioral research studies and neuroimaging investigations have provided a great deal of insight into the nature of memory impairments in DS and their neural correlates, more functional imaging and longitudinal research is needed to examine behavior-brain relations directly in DS. In addition, research employing nuanced memory tasks that have analogues for murine models of DS (such as [143]) will be important for advancing our understanding of the neural and possibly genetic underpinnings of the complex memory difficulties faced by individuals with DS. Such research could shed light on the etiology and developmental unfolding of these impairments and their (predictive) relations to real-world functioning over time. In turn, these investigations may support the development of targeted educational and pharmacological interventions to improve quality of life for those with DS and their families.

Finally, it is important to note that the quality of life and lifespan trajectories of individuals with DS have significantly improved over the past several decades and will hopefully continue to do so (for review, see [10]). Consequently, our understanding of adults with DS today cannot be used to predict the well-being and capabilities of an adult with DS in future years. Thus, it is important to recognize that descriptions of adults with DS are evolving, and thus, research will continue to be needed to provide more accurate descriptions of the capabilities and needs of adults with DS in future generations.

## Additional file

Additional file 1: Table S1. Effect size data descriptions. (DOCX 46 kb)

## Abbreviations

AD: Alzheimer’s disease
CA: Chronological age
DS: Down syndrome
ID: Intellectual disability
MA: Mental age
TD: Typically developing
WS: Williams syndrome
