# Supplementary material for: Memory profiles in Down syndrome across development: a review of memory abilities through the lifespan
Source: J Neurodev Disord. 2018 Jan 29;10:5. doi: 10.1186/s11689-017-9220-y (PMC5789527; doi:10.1186/s11689-017-9220-y)
Supplement: Additional file 1: Table S1. — Effect size data descriptions. (DOCX 46 kb) [file 11689_2017_9220_MOESM1_ESM.docx]

| **Long Term Memory** | | | | | | |
| --- | --- | --- | --- | --- | --- | --- |
| **Study** | **DS Group CA (Yrs)** | **Task** | **Task Descriptions** | **Domain** | | **Effect Size** |
| *Preschool Studies* | | | | | | |
| **Milojevich 2015** [54] | 2.75 | Deferred Imitation | Children observed examiners complete a three-step event sequence twice, and immediately practiced. After a one-month delay, participants were required to perform the event sequence. This measured the participant’s ability to perform target actions in the correct temporal order after the 1 month delay. | **NV** | | **-0.81** |
| **Roberts**  **2015** [35] | 4 | Deferred Imitation | The examiner demonstrated a sequence of three actions to put together a toy rattle. After a 24-hour delay, participants were required to reproduce the sequence. | **NV** | | **0.74** |
|  |  | Object Location Retention | Participants watched an examiner hide a toy item in a toy box. After a 24- hour delay, participants were asked to recall the location of the hidden toy. The outcome variable for this task was the number errors made while searching for the toy^[[1]](#footnote-1)^. | **NV** | | **-0.03** |
| *School Age and Adolescent Studies* | | | | | | |
| **Pennington 2003** [9] | 14 | NEPSY List Learning | Participants were taught a word list of 15 words over 5 trials, and then tested on their ability to recall the word list after a 30-minute delay. The outcome measure was the items recalled across the trials. | **V** | | **-0.51** |
|  |  | Virtual Morris Water Maze | The computerized maze task required participants to use contextual cues to locate a hidden target. The outcome variable measured the amount of time participants spent in the correct region of the maze searching for the target on the probe trial that occurred after 5 test trials. | **NV** | | **-0.80** |
|  |  | CANTAB Pattern Recognition Memory | Participants viewed a series of two blocks of 12 abstract visual patterns on the computer. Then, they completed 12 recognition trials, which required identifying the previously seen pattern. | **NV** | | **-0.56** |
|  |  | CANTAB Paired Associates Learning | The computerized measure that required participants to recall the location of a specific pattern after having viewed the locations of a series of patterns (from 1 to 8 in number). The outcome measure was the number of correctly placed patterns. | **NV** | | **-0.67** |
| **Vicari**  **2005** [62] | 16 | Visual-Object Learning | Participants were presented with pictures of 15 common objects for 5 seconds each. Then they viewed 15 pages with 4 pictures of different variations of the objects and asked to identify the ones they saw before as quickly as possible during three recognition trials. | **NV** | | **-0.91** |
| **Visu-Petra 2007** [120] | 14 | CANTAB Pattern Recognition Memory | Participants viewed a series of patterns on a computer screen and were then presented with pairs of stimuli and were required to identify the previously seen pattern. | **NV** | | **-0.68** |
|  |  | CANTAB Paired Associates Learning | Participants were required to learn associations between a visual pattern and its location. Outcomes was stages completed; more stages indicate greater performance. | **NV** | | **-0.74** |
|  |  | CANTAB Spatial Recognition Memory | Participants are presented with a series of squares in various locations on a computer screen. Then, they are presented with two squares, and must choose the one located in the previously seen location. | **NV** | | **-0.28** |
| *Adult & Old Adult Studies* | | | | | | |
| **Vicari**  **2000** [36] | 21 | Word Recognition Delay | Participants were presented with 15 words and were required to read the word and express their likability of the word. After a ten-minute delay, participants completed a “yes/no” recognition task identifying the 15 previously seen words. Outcome variable was ‘hit rate.’ | **V** | | **-0.90** |
|  |  | Word Recall | Participants completed a word-list learning task in which they had to recall a list of 12 words over 5 learning trials. | **V** | | **-1.40** |
|  |  | Picture Recognition | After a delay, participants were required to identify 14 previously seen pictures out of a total of 28 pictures. | **NV** | | **-0.84** |
|  |  | Spatial Sequences Learning | Participants observed examiners tapping a sequence of spatial locations on a test board (like that used in Corsi span task) that was 2 taps longer than their established short-term memory span. Then the participant had 10 trials to tap the complete sequence correctly. 10 points were earned if this was completed on the first trial and a point was deducted from that for each subsequent trial until the child reached trial 10. | **NV** | | **-2.03** |
| **Davis**  **2014** [67] | 18 | Route Learning Recall | Participants viewed an examiner completing a virtual route with directions. Then participants had up to 8 trials correctly navigate the route. The outcome measure reported here is the number of trials required to learn the route (lower numbers indicated faster learning^1^) | **NV** | | **-1.22** |
| **Short Term Memory** | | | | | |  |
| **Study** | **DS Group CA (Yrs)** | **Task** | **Task Descriptions** | | **Domain** | **Effect Size** |
| *School Age and Adolescent Studies* | | | | | | |
| **Marcell**  **1988 [170]** | 16.75 | Digit Span Recall | Participants immediately recalled a series of digits after hearing a recorded presentation of the digits over a computer speaker (in the auditory freefield condition of this task). | | **V** | **-2.00** |
| **Seung**  **2000** [26] | 16.4 | ITPA Forward Digit Span Recall | Participants immediately recalled a series of digits after hearing an auditory presentation of the digits. | | **V** | **-1.00** |
| **Munir**  **2000 [58]** | 11.17 | Object Location Recall | Adopted from the Kaufman Assessment battery for Children. Participants were required to recall the location of visually presented objects on a sheet of grid paper. | | **NV** | **-1.33** |
|  |  | Digit Span Recall | Participants were required to recall increasingly longer sequences of digits that were orally presented at the rate of one per second. | | **V** | **-1.49** |
| **Jarrold**  **2002** [49] | 14.28 | Corsi Span Recall | Participants observed examiners tapping a sequence of spatial locations on a test board. Participants were required to immediately recall the sequence of locations by tapping the same locations in the correct temporal order. | | **NV** | **0.24** |
|  |  | Digit Recall | Participants immediately recalled a series of digits after hearing a recorded presentation of the digits over a computer | | **V** | **-1.95** |
| **Laws**  **2002** [147] | 11.1 | McCarthy Scales Digit Span Recall | Participants immediately recalled a series of digits after hearing an auditory presentation of the digits and were evaluated on the longest sequence of digits they could recall accurately. | | **V** | **-1.48** |
|  |  | Corsi Span Recall | Participants observed examiners tapping a sequence of spatial locations on a test board. Participants were required to immediately recall the sequence of locations by tapping the same locations in correct temporal order. | | **NV** | **1.43** |
| **Pennington 2003** [9] | 14 | DAS Recall of Digits | Participants immediately recalled a series of digits after hearing an auditory presentation of the digits. | | **V** | **-1.02** |
| **Lanfranchi**  **2004** [115]  Study 1 | 11.75 | Word Span Recall^[[2]](#footnote-2)^ | Participants were presented with an auditory list of 2 to 5 words. Participants were required to immediately recall the list in the same order as presented. | | **V** | **-0.86** |
| **Lanfranchi**  **2004** [115]  Study 2 | 14.5 | Memory for Positions^2^ | Participants were presented with a chessboard (either 3x3 or 4x4). The board was filled with 2 to 4 green squares and participants had 10 seconds to learn the green square locations. Participants were then required to immediately recall the location of the green squares using an empty chessboard. | | **NV** | **0.13** |
|  |  | Pathway Forwards^2^ | Participants were presented with a chessboard (either 3x3 or 4x4), and an examiner moved a frog in a sequenced path across the board (2 to 4 steps). Participants then had to immediately recall the steps taken by the frog. | | **NV** | **0.11** |
| **Cairns**  **2005** [176] | 15.43 | Digit Span Recall | Participants immediately recalled a series of digits after hearing a pre-recorded auditory presentation of the digits. | | **V** | **-1.41** |
| **Visu-Petra**  **2007** [120] | 14 | CANTAB Spatial Span Task | Participants viewed a sequence of squares on a computer screen, which change colors one at a time. The participants were required to immediately recall the sequence by touching the squares in the correct order. | | **NV** | **-0.55** |
| **Frenkel**  **2009** [87] | 12 | Auditory Word Span Task | Word lists of increasing length were aurally presented to participants. Participants were required to the list words in the correct serial order. | | **V** | **-1.20** |
|  |  | Visual Patterns Task | Participants viewed a grid with patterns for two seconds, and then viewed a similar grid with one missing pattern. Participants were required to identify the missing patterns. The number of patterns on each grid increased from 2 to 24 patterns. | | **NV** | **-0.40** |
|  |  | Corsi Block Recall Task | Participants observed examiners tapping a sequence of spatial locations on a test board. Participants were required to recall the sequence of locations by tapping the same locations in correct temporal order. | | **NV** | **0.02** |
| **Lanfranchi**  **2009** **[117]** | 13.1 | Word Span^2^ | Participants were presented with an auditory list of 2 to 5 words. Participants were required to immediately recall the list in the same order as presented. | | **V** | **-1.40** |
|  |  | Path Recall^2^ | Participants were presented with a chessboard (either 3x3 or 4x4), and an examiner moved a frog in a sequenced path across the board (2 to 4 steps). Participants then had to immediately reproduce the series of steps taken by the frog. | | **NV** | **-0.05** |
| **Carretti**  **2010 [183]** | 7.6 | Spatial Location Recall  Structured | For ten seconds, participants were presented with a matrix ( 3x3 to 4x4) with filled cells that formed a pattern. Participants immediately recalled the pattern by indicating the location of the filled cells on a blank grid. | | **NV** | **-1.88** |
|  |  | Spatial Location Recall Unstructured | For ten seconds, participants were presented with a matrix ( 3x3 to 4x4) with randomly arranged filled cells. Participants immediately recalled the pattern by indicating the location of the filled cells on a blank grid. | | **NV** | **-0.54** |
| **Costanzo**  **2013** [90] | 14.5 | Digit Span Recall | Participants immediately recalled a series of digits after hearing an auditory presentation of the digits. | | **V** | **-2.10** |
|  |  | Corsi Span Task | Participants observed examiners tapping a sequence of spatial locations on a test board. Participants were required to immediately recall the sequence of locations by tapping the same locations in correct temporal order. | | **NV** | **-1.44** |
| **Danielsson**  **2016 [91]** | 14.6 | Block Recall | Participants observed examiners tapping a sequence of spatial locations on a test board. Participants were required to immediately recall the sequence of locations by tapping the same locations in correct temporal order. | | **NV** | **0.16** |
|  |  | Word List Recall | Participants were presented with an auditory list of words and required to immediately recall the list in the same order as presented. | | **V** | **-0.74** |
| *Adults and Older Adults Studies* | | | | | | |
| **Marcell**  **1988** [84] | 18.12 | Digit Span Recall | Participants immediately orally recalled a series of digits after hearing an auditory presentation of the digits on this ‘auditory oral’ digit recall task. | | **V** | **-2.00** |
| **Lee**  **2010** [98] | 19.18 | DAS Recall of Digits | Participants immediately recalled a series of digits after hearing an auditory presentation of the digits. | | **V** | **-1.51** |
| **Mosse**  **2011** [193] | 19.5 | Immediate serial recall | Participants heard a recorded word list of four animal names. Then, participants viewed pictures of the four animals and were required to point to or name the pictures of the animals in the correct sequence. | | **V** | **-1.11** |
| **Belacchi**  **2014** [108] | 18.8 | Word Recall^2^ | Participants were presented with an auditory list of 2 to 5 words. Participants were required to immediately recall the list in the same order as presented. | | **V** | **-0.99** |
|  |  | Spatial-simultaneous Recall^2^ | Participants were presented with a chessboard (either 3x3 or 4x4). The board was filled with 2 to 3 green squares and participants had 15 to 20 seconds to learn the green square locations. Participants were then required to immediately recall the location of the green squares using an empty chessboard. | | **NV** | **-0.30** |
| **Working Memory** | | | | | |  |
| **Study** | **DS Group CA (Yrs)** | **Tasks** | **Task Descriptions** | | **Domain** | **Effect Size** |
| *School Age and Adolescent Studies* | | | | | | |
| **Munir**  **2000 [58]** | 11.17 | Backwards Digit Span Recall | Participants were required to recall increasingly longer sequences of digits in the backwards order of the original presentation. Digit sequences were orally presented at the rate of one per second. | | **V** | **-1.41** |
| **Pennington**  **2003** [9] | 14 | Counting Span | Participants were presented with a set of cards (2 to 6 cards) containing blue and yellow dots. Participants were required to count aloud the number of yellow dots on each card. After the entire presentation participants were asked to recall in temporal order, the number of yellow dots on each card. | | **V** | **0.16** |
|  |  | CANTAB Spatial Working Memory | Participants were required to conduct a self-organized search of items to identify ‘virtually’ hidden items on a computer screen. Participants had to recall the locations that they had already searched and avoid returning to them in order to efficiently find the hidden items. The outcome variable of interest was search errors^1^. | | **NV** | **-0.20** |
| **Lanfranchi 2004**  Study 1 [115] | 11.75 | Backward Word Span | Participants were presented with an auditory list of 2 to 5 words. Participants were required to immediately recall the list in the reverse order as presented. | | **V** | **-0.92** |
|  |  | Selective Word Recall | Participants were aurally presented with one to two word lists at a time. After hearing the entire presentation, the participant was required to recall the first word of each list. | | **V** | **-1.38** |
|  |  | Dual Request Word Recall | Participants were presented with an auditory list of 2 to 5 words. Participants were required to recall the first word of the list and to tap the table when the word “ball” was said. | | **V** | **-2.32** |
| **Lanfranchi 2004**  Study 2 [115] | 14.5 | Pathway Backwards | Participants were presented with a chessboard (either 3x3 or 4x4), and an examiner moved a frog in a sequenced path across the board (2 to 4 steps). Participants then had to immediately recall the steps in reverse order. | | **NV** | **-0.24** |
|  |  | Starting Position Selection | Participants were presented with a chessboard (4x4), and an examiner moved a frog in a sequenced path across the board. Level 1 included one path with three steps, and level 2 included one path with four steps. Levels 3, 4, and 5 included two paths, with two, three, and four steps respectively. Then participants were required to recall the starting location of each of the paths learned. | | **NV** | **-0.68** |
|  |  | Dual Request Selective Task | Participants were presented with a chessboard (4x4), that had one red square (out of 16), and an examiner moved a frog in a sequenced path across the board. Participants had to tap the table when a frog jumped onto a red square on the board, and had to recall the starting position of the steps. | | **NV** | **-0.86** |
| **Visu-Petra 2007** [120] | 14.4 | CANTAB Spatial Working Memory | Participants were required to conduct a self-organized search of items to identify hidden items (increasing from 3 to 8 items). Participants had to recall the locations that they had already searched and avoid returning to them in order to efficiently find the hidden items. The outcome variable of interest was errors^1^ | | **NV** | **-1.08** |
| **Lanfranchi 2009** [117] | 13 | Selective Span | Participants were aurally presented with one to two word lists at a time. After hearing the entire presentation, the participant was required to recall the first word of each list. | | **NV** | **-0.6** |
|  |  | Verbal Double Task | Participants were presented with an auditory list of 2 to 5 words. Participants were required to recall the first word of the list and to tap the table when the word “ball” was said. | | **V** | **-1.4** |
|  |  | Starting Position Selection | Participants were presented with a chessboard (4x4), and an examiner moved 1-2 frog(s) in a sequenced path across the board. Participants were instructed to remember the frog’s starting position. The task included four levels. Level 1 included one path with two steps, and Level 2 included one path with three steps. Levels 3, 4, and 5 included two paths with two and three steps. | | **NV** | **-0.40** |
|  |  | Visuospatial Double task | Participants were presented with a chessboard (4x4), that had one red square (out of 16), and an examiner moved a frog in a sequenced path across the board. Participants had to tap the table when a frog jumped onto a red square on the board, and had to recall the starting position of the steps. | | **NV** | **-1.20** |
| **Lanfranchi 2010** [116] | 15.1 | Verbal Dual task | Participants were presented with an auditory list of 2 to 5 words. Participants were required to recall the first word of the list and to tap the table when the word “ball” was said. | | **V** | **-1.03** |
|  |  | Visuospatial Dual task | Participants were presented with a chessboard (4x4), that had one red square (out of 16), and an examiner moved a frog in a sequenced path across the board. Participants had to tap the table when a frog jumped onto a red square on the board, and had to recall the starting position of the steps. The task had four levels of difficulty, including two, three, four, and five step paths. | | **NV** | **-0.95** |
| **Lanfranchi 2012** [119] | 13.7 | Selective Word Recall | Participants were aurally presented with one to two word lists at a time. After hearing the entire presentation, the participant was required to recall the first word of each list. | | **V** | **-0.44** |
|  |  | Verbal Dual task | Participants were presented with an auditory list of 2 to 5 words. Participants were required to recall the first word of the list and to tap the table when the word “ball” was said. | | **V** | **-0.67** |
|  |  | Selective Pathways Recall | Participants were presented with a chessboard (4x4 in size), and an examiner moved a frog in a sequenced path across the board (2 to 4 steps). Participants learned up to two paths at a time. Then participants were required to recall the starting location of each of the paths learned. | | **NV** | **0.04** |
|  |  | Visuospatial Dual Task | Participants were presented with a chessboard (4x4), that had one red square (out of 16), and an examiner moved a frog in a sequenced path across the board. Participants had to tap the table when a frog jumped onto a red square on the board, and had to recall the starting position of the steps. The task had four levels of difficulty, including two, three, four, and five step paths. | | **NV** | **-0.49** |
| **Borella**  **2013** [114] | 14.6 | Verbal Dual Task | Participants were presented with an auditory list of 2 to 5 words. Participants were required to recall the first word of the list and to tap the table when the word “ball” was said. | | **V** | **-0.97** |
| **Costanzo 2013** [90] | 14.5 | Backward Digit Span | After hearing an auditory presentation of a series of digits, participants were required to immediately recall the series of digits in the reverse order of the presentation. | | **V** | **-2.37** |
|  |  | Backward Corsi Span | Participants observed examiners tapping a sequence of spatial locations on a test board. Participants were required to immediately recall the sequence of locations in the reverse order by tapping the same locations in correct temporal order. | | **NV** | **-1.70** |
| *Adult and Older Adult Studies* | | | | | | |
| **Belacchi**  **2014** [108] | 18.8 | First Word List Recall | Participants were aurally presented with one to two word lists at a time. After hearing the entire presentation, the participant was required to recall the first word of each list. | | **V** | **-0.61** |
|  |  | First Stop Position Recall Task | Participants were presented with a chessboard (4x4 in size), and an examiner moved a frog in a sequenced path across the board (2 to 4 steps). Participants learned up to two paths at a time. Then participants were required to recall the starting location of each of the paths learned. | | **NV** | **0.07** |

1. When errors or trials needed to success are reported, lower rather than higher scores denote better performance. Thus, in order to be consistent with other effect sizes calculated for studies in which higher scores denote better performance, the sign was reversed for effect size. So, if the errors were higher in the DS group (which would result in a positive Cohen’s d value), the Cohen’s d was changed to have a negative sign to be consistent with other effect sizes where this denotes that the control group outperformed the DS group. Conversely, if the DS group had fewer errors, the sign was reversed to be positive to denote that they outperformed the controls. [↑](#footnote-ref-1)
2. Note: These authors placed these tasks under the umbrella of ‘working memory’ and distinguished among tasks based on the cognitive load or control required to complete them. However, using the operationalized definitions we provided, these tasks fit under the umbrella of short-term memory. Thus, they are listed in that section of the table. [↑](#footnote-ref-2)
